# Enhancing the Anticancer Activity of Attenuated *Listeria monocytogenes* by Cell Wall Functionalization with “Clickable” Doxorubicin

**DOI:** 10.1021/acschembio.4c00250

**Published:** 2024-09-24

**Authors:** Irene Lepori, Marta Roncetti, Marianna Vitiello, Elisabetta Barresi, Raffaella De Paolo, Paolo Maria Tentori, Caterina Baldanzi, Melissa Santi, Monica Evangelista, Giovanni Signore, Lorena Tedeschi, Claudia Gravekamp, Francesco Cardarelli, Sabrina Taliani, Federico Da Settimo, M. Sloan Siegrist, Laura Poliseno

**Affiliations:** † Institute of Clinical Physiology, National Research Council (CNR-IFC), Pisa 56124, Italy; ‡ Oncogenomics Unit, Core Research Laboratory, ISPRO, Pisa 56124, Italy; § University of Siena, Siena 53100, Italy; ∥ Department of Pharmacy, University of Pisa, Pisa 56126, Italy; ⊥ CISUP-Center for Instrument Sharing, University of Pisa, Pisa 56126, Italy; # Center for Nanotechnology Innovation @NEST, Istituto Italiano di Tecnologia, Pisa 56126, Italy; ∇ NEST-Scuola Normale Superiore, Istituto Nanoscienze, CNR (CNR-NANO), Pisa 56126, Italy; ☆ Fondazione Pisana per la Scienza ONLUS, San Giuliano Terme, Pisa 56017, Italy; □ Department of Microbiology and Immunology, Albert Einstein College of Medicine, New York, New York 10461, United States; ▼ Department of Microbiology, University of Massachusetts, Amherst, Massachusetts 01003-9316, United States; ⊞ Molecular and Cellular Biology Graduate Program, University of Massachusetts, Amherst, Massachusetts 01003-9316, United States

## Abstract

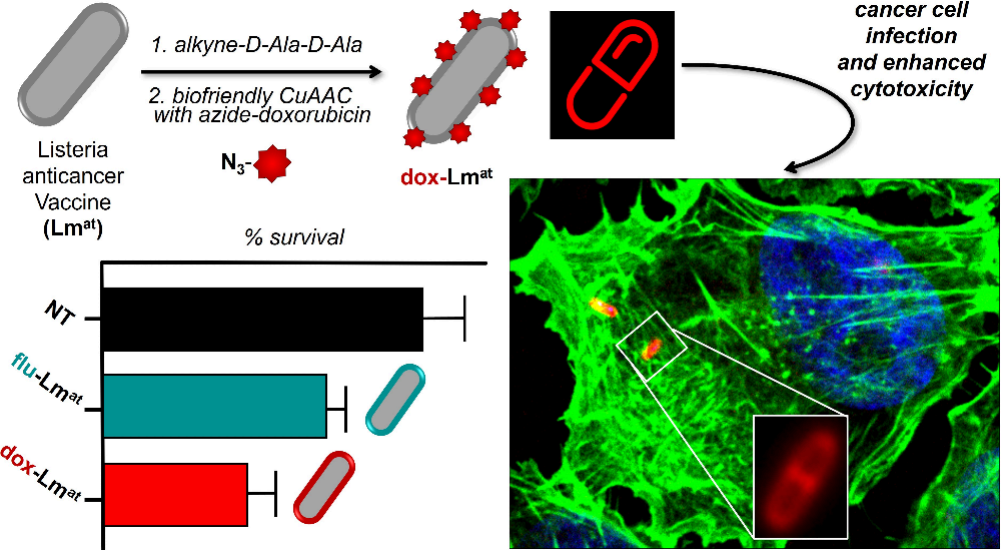

Among bacteria used as anticancer vaccines, attenuated *Listeria monocytogenes* (Lm^at^) stands out, because it spreads from one infected cancer cell to the next, induces a strong adaptive immune response, and is suitable for repeated injection cycles. Here, we use click chemistry to functionalize the Lm^at^ cell wall and turn the bacterium into an “intelligent carrier” of the chemotherapeutic drug doxorubicin. Doxorubicin-loaded Lm^at^ retains most of its biological properties and, compared to the control fluorophore-functionalized bacteria, shows enhanced cytotoxicity against melanoma cells both in vitro and in a xenograft model in zebrafish. Our results show that drugs can be covalently loaded on the Lm^at^ cell wall and pave the way to the development of new two-in-one therapeutic approaches combining immunotherapy with chemotherapy.

## Introduction

Attenuated *Listeria monocytogenes* (Lm^at^) has been widely investigated as an anticancer vaccine, because of its ability to trigger a strong and pleiotropic immune response against primary tumors, as well as metastases. In addition, it can spread from cell to cell, reaching even the deepest, most hypoxic tumor regions. Finally, Lm^at^ does not induce a strong antibody production; therefore, it is suitable for repeated injections.^1,2^

Due to its ability to selectively accumulate inside cancer tissues, Lm^at^ has been largely employed as a platform to deliver different kinds of therapeutic compounds inside the tumor mass.^3,4^ The ease of genetic manipulation has enabled the use of this bacterium as a carrier for nucleic acids,^5^ tumor-associated antigens (TAA),^6^ and prodrug converting enzymes^7^ with anticancer activity. In addition, there have been several successful attempts to combine Lm^at^-mediated immunotherapy with chemotherapy.^8^ The ability of Lm^at^ to deliver clinically relevant, nongenetically encoded molecules has also been exploited by our group, in the form of radiolabeling^9,10^ and noncovalent surface coating of Lm^at^ with antibodies or immunomodulatory molecules.^9,11^ Both strategies were safe for mice and therapeutically effective, leading to a stronger reduction of tumor burden and higher survival rates.

Several approaches have been developed to functionalize the surfaces of bacteria.^12−19^ For Listeria, we and others have shown that the cell wall can be loaded via a combination of (i) metabolic labeling and (ii) bio-orthogonal click chemistry reaction.^20−22^ Our two-step approach consists of (i) metabolic incorporation of azide- or alkyne-bearing d-Alanine probe in the peptidoglycan (PG) stem peptide, followed by (ii) covalent attachment of alkyne- or azide-bearing cargos through copper(I)-catalyzed azide–alkyne cycloaddition (CuAAC). This approach is highly efficient and, if properly tuned, it can be highly biocompatible.^23,24^

Here, we use click chemistry to covalently conjugate the chemotherapeutic drug doxorubicin to the Lm^at^ surface. Our approach has high loading efficiency, is bioorthogonal, and is amenable to both noncleavable and cancer cell-selective cleavable linkers. Having previously demonstrated the effectiveness of Lm^at^ against melanoma cells, both in vitro and in the Braf/Pten melanoma model,^25^ we chose melanoma as biological context and showed that doxorubicin-loaded Lm^at^ has enhanced cytotoxicity against infected melanoma cells, compared to fluorophore-loaded control Lm^at^.

Our loading method broadens the spectrum of tools for the chemical engineering of Lm^at^ and sets up a versatile approach to covalently attach chemotherapeutic small molecules directly on its surface,^26,27^ expanding the bacterium’s utility as an anticancer vaccine.

## Results and Discussion

### Biocompatible and Efficient Lm^at^ Cell Wall Loading with a Fluorophore

The Listeria strain that we used for cell wall loading is XFL-7 Lm^at^-LLO (denoted as Lm^at^, for the sake of brevity). This strain, which has been widely exploited by our research group as a vaccine against breast cancer,^11^ pancreatic cancer^26^ and melanoma,^25^ is characterized by attenuated virulence due to the knockout of the Positive Regulatory Factor A (*prfA*) gene and its reintroduction as an episomal plasmid.^6^

Generation of a bacterium-drug conjugate for therapeutic purposes requires a loading process that is both efficient and able to preserve bacterial viability, ability to interact with host cells, and fitness. We opted for metabolic labeling of the amino acids that compose the stem peptide of PG, followed by covalent attachment of the drug of choice through a click chemistry reaction, because this is a controlled, site-specific approach that, contrary to nonspecific conjugation, allows one to predict and monitor the destiny of the payload. Furthermore, our approach can be adapted to ensure drug release in host cell cytoplasm once the bacterium reaches the tumor mass. The protocol for optimal loading of Lm^at^ cell wall was set up using AF488 fluorophore as cargo.

We compared two of the most common click reactions: the strain-promoted azide–alkyne Cycloaddition (SPAAC)^27^ and the copper(I)-catalyzed azide–alkyne Cycloaddition (CuAAC) reactions.^28^ In Figure S1, we show that, in our experimental setting, the CuAAC reaction is more efficient and able to preserve viability.^29,24^ Then, we chose the commercially available alkyne-modified d-alanine (alkDA), which is expected to be incorporated into the fifth position of the peptidoglycan stem peptide, as a metabolic probe (Figure S2)^20^ and azido-AF488 (az-AF488) as a fluorescent label. Finally, by tuning the components of CuAAC reaction (Figure S3), we established the optimal conditions to obtain AF488-Lm^at^-alkDA with maximal loading efficiency and, at the same time, no observable toxicity (Figure S4). We also observed that the optimized CuAAC reaction is not affected by Lm^at^ genetic background (Figure S5).

**Figure 1:**
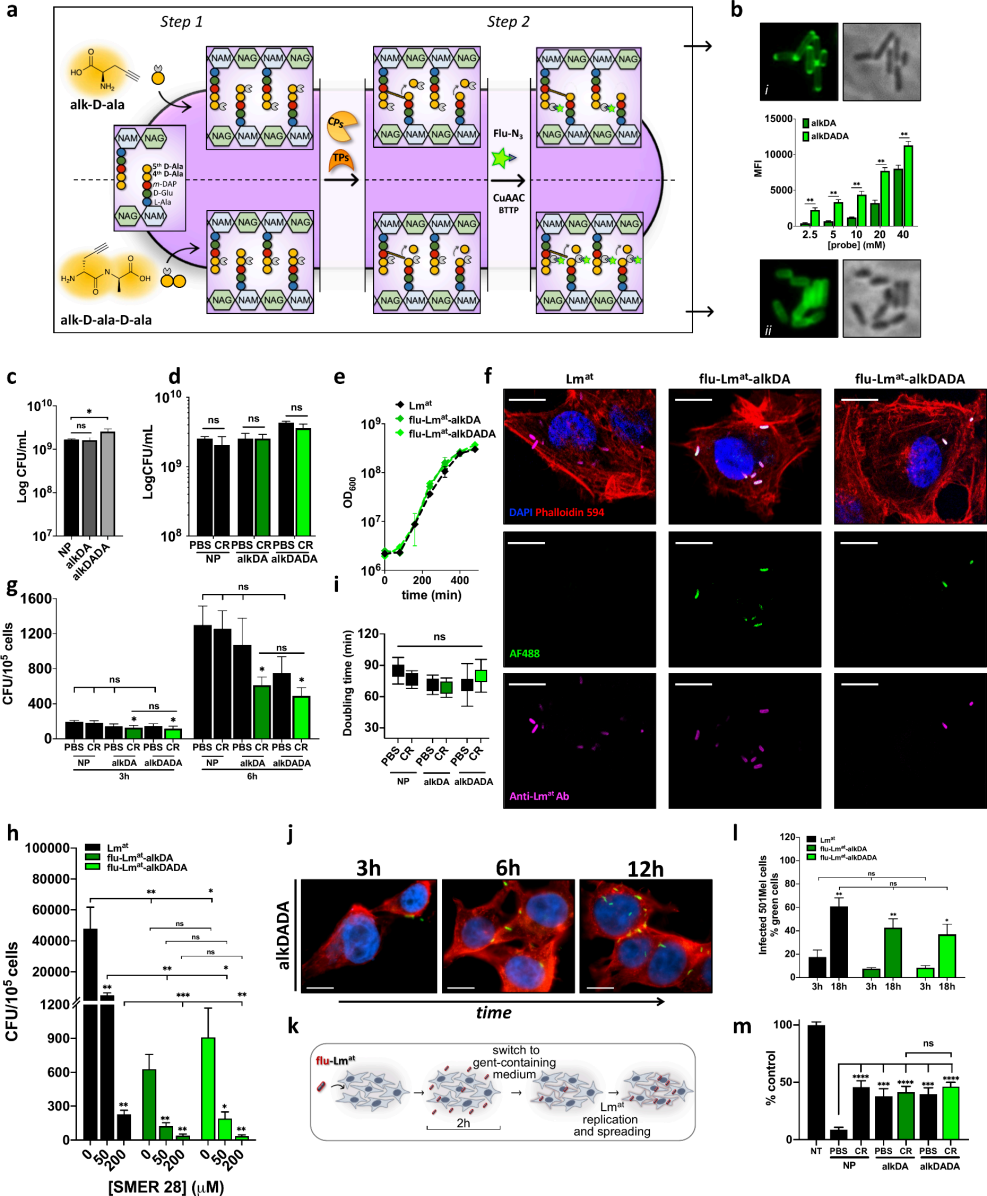
**Generation of flu-Lm^at^ and characterization of its biological features in melanoma cell lines.** (a) Schematic representation of the two-step approach used to functionalize the Lm^at^ cell wall. In the first step, Lm^at^ is incubated with an alkyne-d-alanine probe (alkDA, upper) or an alkyne-d-alanine-d-alanine probe (alkDADA, lower), which result in the metabolic functionalization of the fifth or fourth d-alanine of the PG stem pentapeptide with an alkyne group, respectively. In the second step, the azide-bearing AF488 green fluorophore (az-AF488) is attached to bacterial cell wall via click reaction (CuAAC reaction, using BTTP as the ligand), so that fluorescent Lm^at^ is obtained. When alkDA (but not alkDADA) is used, d,d-carboxypeptidases (CPs) and d,d-transpeptidases (TPs) can remove the alkyne- and/or fluorophore-bearing fifth d-alanine from the PG stem peptide, decreasing loading efficiency. TPs also cross-link the fourth d-alanine to meso-diaminopimelic acid (m-DAP), contributing to confer PG its characteristic 3D meshlike structure. (b) Fluorescence microscope images of bacteria incubated overnight (ON) with 1 mM alkDA probe (i, top) or alkDADA probe (ii, bottom), and MFI of bacteria populations incubated with increasing probe concentrations (middle). (c) Bacteria viability after ON incubation with 40 mM alkDA (dark gray bar) or alkDADA (gray bar) probe. (d) Viability of bacteria subjected to CuAAC reaction, after ON incubation with 40 mM alkDA probe (dark green bar) or alkDADA probe (green bar). For click reaction, the following optimized protocol was used: 25 μM az-AF488, 2.5 mM sodium ascorbate, 20 μM CuSO_4_ and 160 μM BTTP, in PBS buffer, with incubation time set at 10 min (see Figure S4). (e) Proliferation of bacteria incubated ON with 40 mM alkDA or alkDADA probe and then subjected to CuAAC reaction with az-AF488 (flu-Lm^at^-alkDA, dark green line; flu-Lm^at^-alkDADA, green line). Unlabeled Lm^at^ (not incubated with a probe nor subjected to CR) is taken as control (black line). Panels (f)–(h) show infectivity assays of AF488-loaded Lm^at^. (f) Representative confocal images of A375 melanoma cells after 3 h of infection with unlabeled Lm^at^ (left), flu-Lm^at^-alkDA (middle), and flu-Lm^at^-alkDADA (right). Blue denotes DAPI staining of cell nucleus; red denotes staining of actin filaments using Phalloidin 594. Green denotes flu-Lm^at^-alkDA and flu-Lm^at^-alkDADA detected through AF488 green fluorophore. Pink represents a rendering of Lm^at^ staining with primary anti-Listeria antibody and far-red secondary antibody. (g) A375 cells were infected at MOI 100 with bacteria incubated or not with alkDA probe or alkDADA probe and then subjected or not to click reaction. After 1 h of infection, extracellular Lm^at^ was killed by medium replacement with fresh gentamycin-containing medium. At 3 h (left bars) and 6 h (right bars) post-infection, cells were lysed and intracellular Lm^at^ was quantified by plating for CFU. (h) A375 cells were infected at MOI 100 with flu-Lm^at^-alkDA (dark green bars), flu-Lm^at^-alkDADA (green bars) or unlabeled Lm^at^ (not incubated with a probe nor subjected to CR, black bars), in the presence of the indicated concentration of SMER28 inhibitor. After 2 h of infection, extracellular Lm^at^ was killed by medium replacement with fresh gentamycin-containing medium. At 3 h post-infection, cells were lysed and intracellular Lm^at^ was quantified by plating for CFU. Panels (i) and (j) show intracellular replication of AF488-loaded Lm^at^. (i) Bacteria doubling time between 3 h and 6 h was calculated based on the CFU obtained in panel (g). (j) Confocal microscope images of 501 Mel cells infected with flu-Lm^at^-alkDADA at MOI 100. After 1 h of infection, extracellular Lm^at^ was killed by medium replacement with fresh gentamycin-containing medium. The fluorescence images are representative of the increase in the number of intracellular bacteria over time (3, 6, and 12 h post-infection). Legend: blue, DAPI staining of cell nucleus; red, staining of actin filaments using Phalloidin 594; green, flu-Lm^at^-alkDADA detected through AF488 green fluorophore. Panels (k) and (l) show cell-to-cell spreading assays. (k) Schematic representation of the experimental approach. 501 Mel melanoma cells were infected at MOI 50 with Cy5-loaded Lm^at^-alkDA or Lm^at^-alkDADA. After 2 h of infection, extracellular Lm^at^ was killed by medium replacement with fresh gentamycin-containing medium. Then, infected cells were collected at 3 and 18 h post-infection, stained with anti-Listeria primary antibody and Alexa Fluor 488 secondary antibody, and analyzed by flow cytometry to determine the percentage of cells that acquire green fluorescence due to Lm^at^ spreading. (l) Percentage of green 501 Mel cells at 18 vs 3 h post-infection with flu-Lm^at^-alkDA (dark green bars), flu-Lm^at^-alkDADA (green bars), or unlabeled Lm^at^ (not incubated with a probe nor subjected to CR, black bars). (m) Kill rate assay. A375 melanoma cells were infected with AF488-loaded Lm^at^-alkDA or Lm^at^-alkDADA at MOI 2000. At 3 h post-infection, extracellular Lm^at^ was killed by medium replacement with fresh gentamycin-containing medium. At 24 h post-infection, cells were fixed and stained with DAPI to count nuclei by fluorescence microscopy. [Legend: NP, no probe; PBS, no click reaction; CR, click reaction; CFU, colony forming units; MOI, multiplicity of infection; MFI, median fluorescence intensity. Graphs represent the mean ± SEM of at least three independent experiments, performed by using at least two independently functionalized stocks of Lm^at^. Unpaired *t*-test. (*) *p* < 0.05, (**) *p* < 0.01, (***) *p* < 0.001. ns: not statistically significant.]

Next, we aimed to overcome a crucial limitation of d-alanine probes in certain bacteria species including *L. monocytogenes*, namely their susceptibility to d,d-carboxypeptidases like penicillin binding protein 5 (Pbp5), which remove the fifth d-alanine of the stem peptide (Figures S6 and S7a–S7f).^20,30^ We reasoned that a probe designed to install the chemical handle on the fourth d-alanine (instead of the fifth) of the PG stem peptide would be insensitive to Pbp5 activity and would increase PG loading efficiency (Figure S7g).^31^ To this end, we resorted to the alkyne-d-alanine-d-alanine (alkDADA, also known as EDA-DA^31^) probe and compared it with the alkDA probe.^31^ After confirming that both probes properly react with fluorophores containing an azido group (Figure S8a), we proceeded with Lm^at^ loading with az-AF488 (Figure 1a). As expected, the loading increased at the increase of probe concentration and incubation time, yet the alkDADA probe yielded a loading that was consistently higher than that of alkDA (Figure 1b and Figure S8b). Neither probe was toxic for Lm^at^, even after overnight (ON) incubation (Figure 1c), and neither bacteria viability nor proliferation were affected upon CuAAC reaction (see Figures 1d and 1e, as well as Figures S8c–S8g). Importantly, growing Lm^at^ retains its cargo for several generations, although fluorescence is inevitably diluted upon bacterial replication (Figure S8h).

We also investigated whether cell wall loading impacts fluorescent Lm^at^ interaction with host cells, i.e., its ability to infect cancer cells, to spread from cell to cell, and to kill infected cells.

While AF488-loaded Lm^at^ retained its ability to infect A375 melanoma cells (Figure 1f), AF488-loaded Lm^at^ infection was less efficient than that of unlabeled Lm^at^ (Figure 1g). To investigate this phenomenon further, we tested infectivity upon treatment with increasing concentrations of SMER28, a broad-spectrum inhibitor of *L. monocytogenes* penetration within cells.^32,2^ Both unlabeled Lm^at^ (Figure 1h, black bars) and AF488-loaded Lm^at^ (Figure 1h, green bars) showed a dose-dependent reduction in cell penetration (compare 0 vs 50/200 μM SMER28), suggesting that the mechanism(s) used by Lm^at^ to penetrate host cells are dampened but not fundamentally altered by cell wall functionalization. Nevertheless, once inside cancer cells, fluorescent Lm^at^ replicates approximately at the same rate as unlabeled Lm^at^ (Figures 1i and 1j). Although the exact mechanism responsible for the reduction in infectivity remains to be established, we speculate that cell wall functionalization may alter the deformability or accessibility of the heteropolymeric mesh and partially impair surface interactions between bacterial and cancer cells. Additionally, or alternatively, cell wall functionalization may interfere with Lm^at^ protein localization.^33^

After endocytosis and phagosome-escape, Lm^at^ spreads directly from the cytoplasm of one cell into the cytoplasm of another.^2^ To test whether fluorophore loading affects this feature, we monitored the increase in the percentage of infected 501 Mel melanoma cells over time upon the removal of extracellular Lm^at^ (Figure 1k). We conjugated Lm^at^ with azido-Cy5 fluorophore (az-Cy5) and stained intracellular Lm^at^ with anti-Lm antibody coupled with a secondary antibody labeled with AF488 fluorophore. Comparing 3 h and 18 h post-infection, we observed that the increase in the percentage of AF488-positive cells previously infected with unlabeled Lm^at^ (black bars in Figure 1l) and Cy5-positive Lm^at^ (green bars in Figure 1l) is similar. We also performed a direct monitoring of Cy5-positive bacteria-containing 501 Mel melanoma cells. As shown in Figure S9a, we observed that the percentage of such cells increases over time only in the case of the spreading-competent Cy5-Lm^at^-alkDA strain, not in the case of Cy5-Lm^at^-OVA-alkDA strain, which is avirulent, because of it being unable to escape the phagosome and, thus, spread cell to cell.^25^ Finally, we observed that the increase over time in the percentage of Cy5-positive bacteria-containing cells has similar trend upon infection with Cy5-loaded Lm^at^-alkDA and Lm^at^-alkDADA (Figure S9b). All together, these results attest that fluorophore-loaded Lm^at^ fully retains its ability to spread cell to cell.

The cytotoxicity exerted by fluorescent Lm^at^ against melanoma cells was measured using a kill rate assay. AF488-loaded Lm^at^ retained its ability to kill A375 cells, but cell wall functionalization has a negative impact on this biological feature, which becomes evident at high MOI (compare the results obtained with MOI 200 (Figure S9c) with those obtained with MOI 2000 (Figure 1m)). The lower cytotoxicity of AF488-loaded Lm^at^-alkDA and Lm^at^-alkDADA (green bars in Figure 1m) is consistent with their impaired infectivity (green bars in Figure 1g). However, bacteria only incubated with the probes (alkDA-PBS bar and alkDADA-PBS bar in Figure 1m), or only subjected to the CuAAC reaction (NP-CR bar in Figure 1m), show reduced cytotoxicity as well. Although statistical significance is not reached, incubation with the two probes does decrease the infectivity (alkDA-PBS bar and alkDADA-PBS bar in Figure 1g, 6 h). This suggests that, when present in abundance within the cell wall, even the minor chemical modification represented by the alkyne group can affect Lm^at^ biological properties, compromising its ability to interact with and later kill host cells. Conversely, the decreased cytotoxicity of Lm^at^ only subjected to a click reaction might be a consequence of the presence of copper(I) in the reaction mix.

In summary, we carefully optimized metabolic labeling and CuAAC reaction to achieve high levels of Lm^at^ cell wall loading without compromising bacterial viability and proliferation. However, a decrease in infectivity, with a consequent decrease in cytotoxicity, are observed. Given the superior loading efficiency compared to alkDA, we chose alkDADA probe to optimize bacterial cell wall loading with a drug. Therefore, in the experiments aimed at assessing the increased cytotoxicity of drug-loaded Lm^at^, we used fluorophore-loaded Lm^at^-alkDADA as a control.

### Doxorubicin Conjugation Increases Lm^at^ Cytotoxicity against Melanoma Cells

The optimization of the two-step loading approach with fluorophores enabled precise and quantitative characterization of each variable involved in the cell wall loading of Lm^at^. However, the physicochemical properties of individual small molecules require some tailoring of the conjugation protocol. More specifically, the optimal conditions defined for the first step (i.e., the metabolic incorporation of the probe) can be applied irrespective of the chosen cargo, while the second step (i.e., the click reaction) requires small molecule-tailored optimization. Several characteristics of the chosen drug, such as water solubility, steric hindrance, and polarity, may in fact affect the accessibility of the azido-modified drug to the alkyne group embedded in the thick, meshlike layer of Gram-positive peptidoglycan.^34,35^

As a proof-of-concept drug to functionalize Lm^at^, we chose doxorubicin (dox). This drug is well-known to cause cytotoxicity due to nuclear accumulation and DNA damage,^36^ while its intrinsic red fluorescence facilitates the assessment of the efficiency of the functionalization process. We also investigated two different chemical linkers to attach doxorubicin onto the Lm^at^ surface. The azidoacetic linker (Figure S10a) is small and uncleavable. It was chosen for its compact size, which is expected to minimize the steric hindrance and facilitate incorporation into the peptidoglycan mesh. The az-VC linker contains the azido group (az), a PEG_4_ spacer attached to a Valine-Citrulline dipeptide (VC) and a self-immolative *para*-aminobenzyl carbamate (PABC) spacer (Figure S11a). Although longer and bulkier than the azidoacetic linker, the az-VC linker allows the specific cleavage of Valine-Citrulline dipeptide by Cathepsin proteases, which are overexpressed in cancer tissues.^37^ This feature, together with the presence of the self-immolative spacer, is expected to enable release of native doxorubicin inside infected cells.^38^

First, we conjugated doxorubicin with the commercially available azidoacetic linker, obtaining az-dox (Figures S10b–S10d), while an az-VC linker conjugated with doxorubicin was purchased from a commercial source (az-VC-dox). Then, we tested the biological effects of the linker-dox conjugates on melanoma cells. We found that even the small 3′-N-modification on the aminoglycoside portion of the drug leads to the loss of the ability of az-dox to accumulate inside nuclei (Figure S11b, compare panel iii with panel iv), with a consequent decrease in cytotoxicity (Figure S11c, compare the third and fourth bar). As expected, az-VC-dox was totally unable to accumulate in cell nuclei (Figure S11b, panel v) and showed an even lower cytotoxicity (Figure S11c, fifth bar). However, both nuclear localization and cytotoxicity were fully restored after incubation with acidified cell lysate containing active Cathepsins that cleave the VC linker and allow the release of doxorubicin in its native form (Figure S11b, panel vi and Figure S11c, sixth bar). As a further indication of Cathepsin-mediated release of native doxorubicin, az-VC-dox showed higher toxicity in SK-Mel-28 cells, which express Cathepsin B at higher levels, compared to A375 cells (Figures S11d and S11e).

We then proceeded with the optimization of Lm^at^ surface functionalization, tailored for the doxorubicin drug. First, we optimized experimental conditions such that the drug is not toxic for Lm^at^. A long incubation under active replication conditions (30–120 min, 37 °C, BHI medium) is, in fact, toxic at doxorubicin concentrations as low as 20 μM (Figure 12a). However, a short incubation in the bacteriostatic conditions used for CuAAC reaction (10 min, RT, PBS buffer) is not associated with toxicity at doxorubicin concentrations as high as 500 μM (Figure S12b). Next, we addressed poor doxorubicin solubility/stability in a CuAAC reaction buffer (PBS, Figure S12c), which would severely impact efficiency and specificity of the conjugation with bacterial surface. We identified the physiological solution (0.9% w/v NaCl) as the best-performing reaction solvent (Figures S12d and S12e). Moreover, we found that a short incubation in the bacteriostatic conditions used for CuAAC reaction was not associated with toxicity using up to 40% dimethyl sulfoxide (DMSO) as a cosolvent (10 min, RT, PBS buffer, Figures S12f and S12g).

Overall, we defined the following as optimal reaction conditions that ensure maximal loading efficiency: 0.9% w/v NaCl solution as reaction solvent; 25% DMSO as a cosolvent; a 3-fold increase in click-reagent concentrations (7.5 mM sodium ascorbate, 60 μM CuSO_4_ and 480 μM BTTP), compared to the protocol used to obtain fluorescent Lm^at^-alkDADA (2.5 mM sodium ascorbate, 20 μM CuSO_4_ and 160 μM BTTP, see above); 200 μM az-dox or az-VC-dox (instead of 25 μM az-fluorophore).

**Figure 2:**
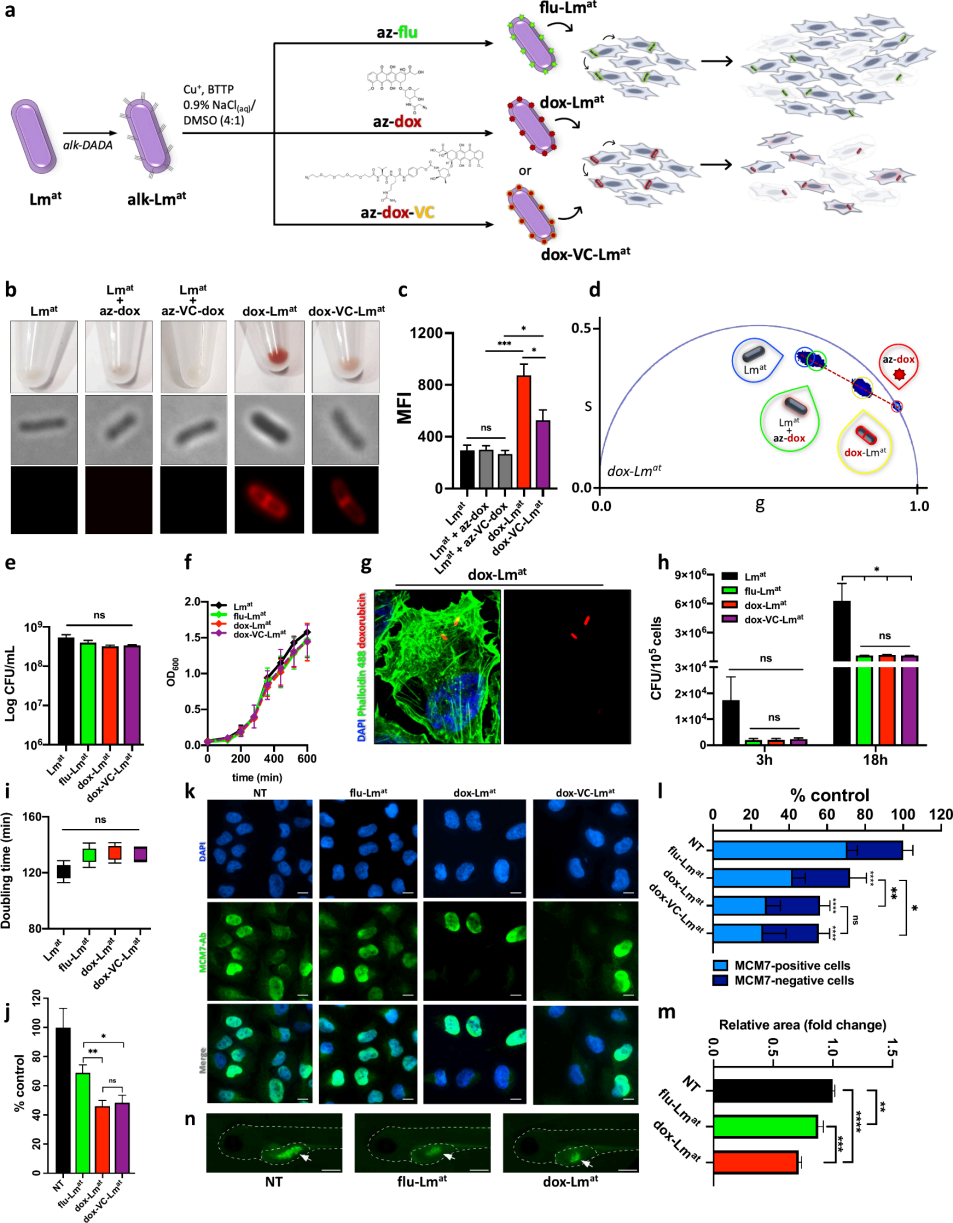
**dox-Lm^at^ shows enhanced anticancer potential in melanoma cell lines.** (a) Schematic representation of the experimental design used to functionalize Lm^at^ with doxorubicin and investigate whether dox-loaded Lm^at^ has enhanced cytotoxicity in vitro. Once preincubated with alkDADA, Lm^at^ is loaded with azide-bearing molecules (az-ATTO740 (az-flu), az-doxorubicin (az-dox) or az-VC-doxorubicin (az-VC-dox)) via CuAAC reaction to generate flu-Lm^at^, dox-Lm^at^, and dox-VC-Lm^at^, respectively. For click reaction, the following optimized protocol was used: 5 μM az-ATTO740, 200 μM az-dox, or 200 μM az-VC-dox; 7.5 mM sodium ascorbate, 60 μM CuSO_4_ and 480 μM BTTP; 0.9% w/v NaCl in water as reaction solvent; 25% DMSO as a cosolvent. After infection with flu-Lm^at^, melanoma cells show decreased viability due to bacteria intrinsic cytotoxicity, which is enhanced when dox-Lm^at^ or dox-VC-Lm^at^ are used instead. (b) Pictures of bacterial cell pellets (top) and fluorescence microscope images (bottom) of: untreated Lm^at^; Lm^at^ not metabolically labeled with the probe, but subjected to CuAAC reaction with az-dox (Lm^at^ + az-dox) or az-VC-dox (Lm^at^ + az-VC-dox); dox-Lm^at^; dox-VC-Lm^at^. (c) Quantification by flow cytometry of the MFI of the samples treated as in panel (b). (d) FLIM phasors plot of dox-Lm^at^. The phasor populations of the different samples lie on different regions of the plot. From left to right: untreated Lm^at^ (blue teardrop); Lm^at^ not metabolically labeled with the probe but subjected to CuAAC reaction with az-dox (green teardrop); dox-Lm^at^ (yellow teardrop); az-dox (red teardrop). (e, f) Viability (panel (e)) and proliferation (panel (f)) of untreated Lm^at^ (black), flu-Lm^at^ (green), dox-Lm^at^ (red), and dox-VC-Lm^at^ (purple). Panels (g) and (h) show infectivity assays. (g) Representative confocal microscope images of A375 cells after 3 h of infection with dox-Lm^at^. Blue denotes DAPI staining of cell nucleus. Green denotes staining of actin filaments using Phalloidin 488. Red denotes dox-Lm^at^. (h) A375 cells were infected at MOI 100 with untreated Lm^at^ (black), flu-Lm^at^ (green) dox-Lm^at^ (red), and dox-VC-Lm^at^ (purple). After 2 h of infection, extracellular Lm^at^ was killed by medium replacement with fresh gentamycin-containing medium. At 3 h (left bars) and 18 h (right bars) post-infection, cells were lysed and intracellular Lm^at^ was quantified by plating for CFU. (i) Intracellular replication of bacteria. Bacteria doubling time between 3 h and 18 h was calculated based on the CFU obtained in (h) for Lm^at^ (black), flu-Lm^at^ (green), dox-Lm^at^ (red), and dox-VC-Lm^at^ (purple). (j) Kill rate assay. A375 melanoma cells were infected with flu-Lm^at^, dox-Lm^at^ or dox-VC-Lm^at^ at MOI 2000. At 3 h post-infection, extracellular Lm^at^ was killed by medium replacement with fresh gentamycin-containing medium. At 48 h post-infection, cells were fixed and stained with DAPI to count nuclei by fluorescence microscopy. Panels (k) and (l) show the proliferation status of A375 melanoma cells infected with flu-Lm^at^, dox-Lm^at^, or dox-VC-Lm^at^ at MOI 1000. After 3 h post-infection, extracellular Lm^at^ was killed by medium replacement with fresh gentamycin-containing medium. At 48 h post-infection, cells were stained with anti-MCM7 antibody and proliferative vs nonproliferative cells were counted based on the presence vs absence of MCM7 nuclear staining. Representative microscope images (panel (k)) and quantification (panel (l)) of proliferative and nonproliferative A375 cells after infection with flu-Lm^at^, dox-Lm^at^, or dox-VC-Lm^at^. Blue denotes DAPI; green denotes anti-MCM7 antibody. Panels (m) and (n) show the area of cancer cell mass developed in a xenograft model in zebrafish embryos. eGFP-expressing A375-PIG cells, previously infected with flu-Lm^at^ or dox-Lm^at^ at MOI 1000 for 2 h, were injected in 48 hpf embryos. [Here, and throughout, hpf stands for hours post-fertilization.] Then, embryos were allowed to grow for additional 48 h. At the end of this period, the area of green cancer cell mass was quantified. (m) Results of area quantification; at least 100 embryos were injected per experimental condition. (n) Representative pictures of 96 hpf embryos that, 48 h earlier, were injected with A375-PIG cells uninfected (left), infected with flu-Lm^at^ (middle), or infected with dox-Lm^at^ (right). The shape of the embryo and the perimeter of the injection site (yolk sac) are highlighted with a white dotted line, while the mass of cancer cells within the yolk sac (indicated with a white arrow) shows a green fluorescence signal. Scale bar = 300 μm. [Legend: NT, untreated cells; NP, no probe; PBS, no click reaction; CR, click reaction; CFU, colony forming units; MOI, multiplicity of infection; MFI, median fluorescence intensity. Graphs represent the mean ±SEM of at least three independent experiments, performed by using at least two independently functionalized stocks of Lm^at^.] Unpaired *t*-test (in vitro assays), Kruskal–Wallis test (Dunn’s multiple comparisons test, xenograft assay). (*) *p* < 0.05, (**) *p* < 0.01, (***) *p* < 0.001, (****) *p* < 0.0001. ns, not statistically significant.]

Since our reaction conditions for doxorubicin loading were different from those used to load fluorophores, we reinvestigated bacterial physiology after cell wall functionalization with both the azidoacetic linker and the cleavable az-VC linker. dox-Lm^at^-alkDADA and dox-VC-Lm^at^-alkDADA were generated (denoted as dox-Lm^at^ and dox-VC-Lm^at^, respectively, for the sake of brevity), while ATTO740-loaded Lm^at^-alkDADA (flu-Lm^at^, for the sake of brevity; see Figure 2a) was used as control. ATTO740 fluorophore was chosen for its excitation/emission spectrum that does not overlap with blue, green, and red fluorescence channels.

The effective conjugation of doxorubicin to Lm^at^ was detectable by eye, as a bacterial pellet color change (Figure 2b, top) and was confirmed by fluorescence microscopy (Figure 2b, bottom), flow cytometry (Figure 2c), and fluorescence lifetime imaging (FLIM, Figure 2d). In particular, the phasor approach to FLIM data allowed us to graphically assign a lifetime signature to any fluorescence species, including weak fluorophores like doxorubicin, and autofluorescent biological entities like bacteria.^39,40^Figure 2d shows the phasors plot of dox-Lm^at^ and related controls. As expected, the phasor population generated by dox-Lm^at^ (yellow teardrop) lies on the segment that connects the phasors of the two unconjugated species (namely, az-dox (red teardrop) and untreated Lm^at^ (blue teardrop)). In addition, when Lm^at^ is not metabolically labeled with the probe, but is still subjected to CuAAC reaction with az-dox, it generates a phasor population that lies very close to that of untreated Lm^at^ (green teardrop). This result strongly suggests that az-dox is conjugated to Lm^at^ via alkDADA, otherwise, unable to adsorb on Lm^at^, it would be washed off as unreacted excess. Finally, we extracted doxorubicin from bacterial cell wall through enzymatic digestion with a mutanolysin/lysozyme mix and used a calibration curve to quantify the amount of drug loaded on dox-Lm^at^ vs dox-VC-Lm^at^. In agreement with visual inspection, fluorescence microscopy and flow cytometry, we found that the loading of dox-Lm^at^ is ∼10-fold higher than that of dox-VC-Lm^at^ (Figures S13a–S13c). By exposing dox-VC-Lm^at^ to acidified cell lysate containing active Cathepsins, we also confirmed that the VC-dox linker remains cleavable upon loading onto the Listeria cell wall (Figure S13d).

After verifying that dox-loaded Lm^at^ retains viability (Figure 2e) and proliferative activity (Figure 2f), we investigated its ability to infect A375 melanoma cells.

As expected, there was a significant reduction in infectivity, but it was comparable across bacteria loaded with all three different cargos (Figures 2g and 2h). The replication rate inside cancer cells was similar for Lm^at^ loaded with the three different cargos (Figure 2i).

To assess the anticancer potential of dox-loaded Lm^at^, we performed a kill rate assay on A375 cells. As shown in Figure 2j, we found that infection with both dox-Lm^at^ and dox-VC-Lm^at^ causes a significant reduction in cell number, compared to flu-Lm^at^. We also noticed that such a decrease is associated with a decrease in cell proliferation rather than an increase in dead cells. Therefore, we explored the replication state of infected cells by investigating the MCM7 protein. This well-known marker is recruited in the DNA replication machinery during active proliferation, and thus its localization switches from cytoplasmic to nuclear only in cells that are actively replicating.^41^ As shown by microscope images (Figure 2k) and related quantitation (Figure 2l), A375 cells infected with both dox-Lm^at^ and dox-VC-Lm^at^ show a significantly lower percentage of replicating, MCM7-positive cells compared to the ones infected with flu-Lm^at^.

We speculate that dox-Lm^at^ and dox-VC-Lm^at^ block melanoma cell proliferation at the same rate, despite the distinct strengths and weaknesses of the linkers. The small azidoacetic linker strongly favors Lm^at^ PG functionalization (Figure 2c) but lacks a release system for the native drug, leaving the fate of the drug attached to Lm^at^ surface to nonspecific mechanisms of drug release such as bacterial PG remodeling and host degradative enzymes.^42^ As a consequence, doxorubicin conjugated to Lm^at^ via the azidoacetic linker is likely released in a 3′-N-modified form that cannot accumulate in cell nuclei (Figure S11b), hence its diminished toxic potential (Figures S11c and S11e). Conversely, the size of az-VC linker decreases conjugation efficiency (Figure 2c), but on the other hand ensures that the drug released inside melanoma cells is in its native form and can accumulate inside the nuclei (Figure S11b) and fully exert its cytotoxic potential (Figures S11c and S11e).

The anticancer potential of dox-loaded Lm^at^ was assessed in vivo as well, using a xenograft model in zebrafish. A375 cells previously infected with flu-Lm^at^ or dox-Lm^at^ at MOI 1000 were injected into the yolk sac of 48 hpf zebrafish embryos. [Note: hpf = hours post-fertilization.] Then, 48 h later, the tumor area was measured and, consistently with in vitro results, we found that dox-Lm^at^ is a stronger inhibitor of tumor growth, compared to flu-Lm^at^ (Figures 2m and 2n).

## Concluding Remarks and Future Perspectives

The use of bacteria as immunotherapeutic agents has gained momentum in recent decades, mainly because these organisms can accumulate selectively in the cancer microenvironment but also because they are straightforward to manipulate and inexpensive. Therefore, immunotherapeutic bacteria are a sustainable option, especially for low-medium income countries.^43^ With the aim to further increase their anticancer activity, bacteria have also been exploited as delivery platforms.^44,45^ Many strategies have been developed that enable bacteria to express genetically encoded, therapeutically useful oligonucleotides, peptides, or proteins. Here, we develop a generalizable approach for functionalizing the surface of an immunotherapeutic bacterium with small molecules.

The Lm^at^ life cycle makes the organism a particularly attractive candidate for small-molecule functionalization. After host-cell-receptor-mediated endocytosis, the ability of Lm^at^ to escape the phagosome gives a great advantage to surface-attached small molecules, as they are delivered directly to the cytoplasm. Furthermore, cell-to-cell spreading allows the molecules not only to be selectively carried into the cancer microenvironment, but also to overcome the major barriers represented by the highly impermeable tumor mass, without relying on tumor vascularization, which is poor, and passive diffusion from one cell to another, which is slow and inefficient.^46^ Finally, selective tropism for tumor sites is due to the fact that they are immunosuppressed, while it is independent of their genetic makeup. In other words, Lm^at^ does not need to be customized to reach a specific cancer type.^47−50^

The metabolic labeling/click chemistry protocol that we have refined here consists of two steps, which were both optimized, so that loading is maximized and, at the same time, viability and proliferation of loaded Lm^at^ are fully preserved: (i) the incorporation of alkDADA in peptidoglycan stem peptide; (ii) the covalent attachment of an azide-bearing cargo through CuAAC reaction. The two-step protocol enables robust cell wall incorporation (the alkyne reactive handle is compact and well-tolerated), as well as modular conjugation of any azide-bearing, small molecule with therapeutic potential. Furthermore, we showed that drug release in the cancer microenvironment can be enhanced by including a release system.

In light of the results obtained with noncovalently coated Lm^at^,^9,11^ we expect that drug-loaded Lm^at^ is well-tolerated when systemically administered in vivo. Based on the fact that (i) in vitro, intracellular Lm^at^ shows persistent labeling and (ii) in vivo, it is carried by myeloid-derived suppressor cells (MDSCs),^51,26^ reaching the tumor microenvironment within a few hours from injection,^9,25^ we also assume that Lm^at^ will still be loaded with the drug when it gets to its intended destination. There, on-site drug release mechanisms (the physiological bacterial clearance and/or the action of intracellular as well as extracellular proteases) should enable specific and effective cancer cell targeting.^52^ Since Lm^at^ does not trigger a strong humoral reaction (the small amount of antibody produced is not sufficient to protect against a reinfection^53^), we also speculate that the loaded bacterium is suitable for repeated injections that ensure steady drug delivery to the tumor microenvironment. Finally, the in vivo setting will allow us to appreciate the immunogenicity of loaded Lm^at^, which is crucial to assess whether our approach indeed combines chemotherapy (doxorubicin) with immunotherapy (Lm^at^). Interestingly, Lm^at^ itself is cytotoxic for cancer cells by causing ROS production,^25,54^ while doxorubicin itself displays immunogenic properties.^55^ Therefore, the cell-autonomous and noncell autonomous anticancer effects of dox-loaded Lm^at^ are expected to be highly pleiotropic and, hence, powerful.

In conclusion, our work describes a new approach for chemical engineering of the Lm^at^ surface and opens new possibilities for combination therapies in cancer treatment.

## Supporting Information Available

Details regarding the setup of click reactions, the biological properties of loaded listeria, and the chemical and biological features of doxorubicin as cargo (Supplementary Figures 1–13). It also contains the description of all experimental protocols (Supplementary Methods). (PDF)

## References

[ref1] Flickinger J. C. Jr.; Rodeck U.; Snook A. E. Listeria Monocytogenes as a Vector for Cancer Immunotherapy: Current Understanding and Progress. Vaccines (Basel) 2018, 6 (3), 48 10.3390/vaccines6030048. 30044426 PMC6160973

[ref2] Radoshevich L.; Cossart P. Listeria Monocytogenes: Towards a Complete Picture of Its Physiology and Pathogenesis. Nat. Rev. Microbiol 2018, 16 (1), 32–46. 10.1038/nrmicro.2017.126. 29176582

[ref3] Forbes N. S.; Coffin R. S.; Deng L.; Evgin L.; Fiering S.; Giacalone M.; Gravekamp C.; Gulley J. L.; Gunn H.; Hoffman R. M.; Kaur B.; Liu K.; Lyerly H. K.; Marciscano A. E.; Moradian E.; Ruppel S.; Saltzman D. A.; Tattersall P. J.; Thorne S.; Vile R. G.; Zhang H. H.; Zhou S.; McFadden G. White Paper on Microbial Anti-Cancer Therapy and Prevention. J. Immunother. Cancer 2018, 6 (1), 78 10.1186/s40425-018-0381-3. 30081947 PMC6091193

[ref4] Zhou S.; Gravekamp C.; Bermudes D.; Liu K. Tumour-Targeting Bacteria Engineered to Fight Cancer. Nat. Rev. Cancer 2018, 18 (12), 727–743. 10.1038/s41568-018-0070-z. 30405213 PMC6902869

[ref5] van Pijkeren J. P.; Morrissey D.; Monk I. R.; Cronin M.; Rajendran S.; O’Sullivan G. C.; Gahan C. G. M.; Tangney M. A Novel *Listeria Monocytogenes* -Based DNA Delivery System for Cancer Gene Therapy. Hum. Gene Ther. 2010, 21 (4), 405–416. 10.1089/hum.2009.022. 20105075

[ref6] Wood L. M.; Paterson Y. Attenuated Listeria Monocytogenes: A Powerful and Versatile Vector for the Future of Tumor Immunotherapy. Front. Cell Infect. Microbiol. 2014, 4, 51 10.3389/fcimb.2014.00051. 24860789 PMC4026700

[ref7] Stritzker J.; Pilgrim S.; Szalay A. A.; Goebel W. Prodrug Converting Enzyme Gene Delivery by *L. monocytogenes*. BMC Cancer 2008, 8 (1), 94 10.1186/1471-2407-8-94. 18402662 PMC2329648

[ref8] Oladejo M.; Paterson Y.; Wood L. M. Clinical Experience and Recent Advances in the Development of Listeria-Based Tumor Immunotherapies. Front. Immunol. 2021, 12, 642316 10.3389/fimmu.2021.642316. 33936058 PMC8081050

[ref9] Quispe-Tintaya W.; Chandra D.; Jahangir A.; Harris M.; Casadevall A.; Dadachova E.; Gravekamp C. Nontoxic Radioactive Listeria(at) Is a Highly Effective Therapy against Metastatic Pancreatic Cancer. Proc. Natl. Acad. Sci. U. S. A. 2013, 110 (21), 8668–8673. 10.1073/pnas.1211287110. 23610422 PMC3666740

[ref10] Chandra D.; Selvanesan B. C.; Yuan Z.; Libutti S. K.; Koba W.; Beck A.; Zhu K.; Casadevall A.; Dadachova E.; Gravekamp C. 32-Phosphorus Selectively Delivered by Listeria to Pancreatic Cancer Demonstrates a Strong Therapeutic Effect. Oncotarget 2017, 8 (13), 20729–20740. 10.18632/oncotarget.15117. 28186976 PMC5400540

[ref11] Singh M.; Quispe-Tintaya W.; Chandra D.; Jahangir A.; Venkataswamy M. M.; Ng T. W.; Sharma-Kharkwal S.; Carreno L. J.; Porcelli S. A.; Gravekamp C. Direct Incorporation of the NKT-Cell Activator Alpha-Galactosylceramide into a Recombinant Listeria Monocytogenes Improves Breast Cancer Vaccine Efficacy. Br. J. Cancer 2014, 111 (10), 1945–1954. 10.1038/bjc.2014.486. 25314062 PMC4229631

[ref12] Zoaby N.; Shainsky-Roitman J.; Badarneh S.; Abumanhal H.; Leshansky A.; Yaron S.; Schroeder A. Autonomous Bacterial Nanoswimmers Target Cancer. J. Controlled Release 2017, 257, 68–75. 10.1016/j.jconrel.2016.10.006. PMC6679715 27744036

[ref13] Xie S.; Zhao L.; Song X.; Tang M.; Mo C.; Li X. Doxorubicin-Conjugated Escherichia Coli Nissle 1917 Swimmers to Achieve Tumor Targeting and Responsive Drug Release. J. Controlled Release 2017, 268, 390–399. 10.1016/j.jconrel.2017.10.041. 29101053

[ref14] Xie S.; Chen M.; Song X.; Zhang Z.; Zhang Z.; Chen Z.; Li X. Bacterial Microbots for Acid-Labile Release of Hybrid Micelles to Promote the Synergistic Antitumor Efficacy. Acta Biomater. 2018, 78, 198–210. 10.1016/j.actbio.2018.07.041. 30036720

[ref15] Xie S.; Xia T.; Li S.; Mo C.; Chen M.; Li X. Bacteria-Propelled Microrockets to Promote the Tumor Accumulation and Intracellular Drug Uptake. Chem. Eng. J. 2020, 392, 123786 10.1016/j.cej.2019.123786.

[ref16] Moreno V. M.; Álvarez E.; Izquierdo-Barba I.; Baeza A.; Serrano-López J.; Vallet-Regí M. Bacteria as Nanoparticles Carrier for Enhancing Penetration in a Tumoral Matrix Model. Adv. Mater. Interfaces 2020, 7 (11), 1901942 10.1002/admi.201901942. 33154882 PMC7116290

[ref17] Zheng D.-W.; Chen Y.; Li Z.-H.; Xu L.; Li C.-X.; Li B.; Fan J.-X.; Cheng S.-X.; Zhang X.-Z. Optically-Controlled Bacterial Metabolite for Cancer Therapy. Nat. Commun. 2018, 9 (1), 1680 10.1038/s41467-018-03233-9. 29700283 PMC5920064

[ref18] Wu F.; Liu J. Decorated Bacteria and the Application in Drug Delivery. Adv. Drug Delivery Rev. 2022, 188, 114443 10.1016/j.addr.2022.114443. 35817214

[ref19] Zeng X.; Li P.; Yan S.; Liu B.-F. Reduction/PH-Responsive Disassemblable MOF-Microbial Nanohybrid for Targeted Tumor Penetration and Synergistic Therapy. Chem. Eng. J. 2023, 452, 139517 10.1016/j.cej.2022.139517.

[ref20] Siegrist M. S.; Whiteside S.; Jewett J. C.; Aditham A.; Cava F.; Bertozzi C. R. (d)-Amino Acid Chemical Reporters Reveal Peptidoglycan Dynamics of an Intracellular Pathogen. ACS Chem. Biol. 2013, 8 (3), 500–505. 10.1021/cb3004995. 23240806 PMC3601600

[ref21] Siegrist M. S.; Aditham A. K.; Espaillat A.; Cameron T. A.; Whiteside S. A.; Cava F.; Portnoy D. A.; Bertozzi C. R. Host Actin Polymerization Tunes the Cell Division Cycle of an Intracellular Pathogen. Cell Rep. 2015, 11 (4), 499–507. 10.1016/j.celrep.2015.03.046. 25892235 PMC4417095

[ref22] Kelliher J. L.; Grunenwald C. M.; Abrahams R. R.; Daanen M. E.; Lew C. I.; Rose W. E.; Sauer J.-D. PASTA Kinase-Dependent Control of Peptidoglycan Synthesis via ReoM Is Required for Cell Wall Stress Responses, Cytosolic Survival, and Virulence in Listeria Monocytogenes. PLoS Pathog. 2021, 17 (10), e1009881 10.1371/journal.ppat.1009881. 34624065 PMC8528326

[ref23] García-Heredia A.; Pohane A. A.; Melzer E. S.; Carr C. R.; Fiolek T. J.; Rundell S. R.; Lim H. C.; Wagner J. C.; Morita Y. S.; Swarts B. M.; Siegrist M. S. Peptidoglycan Precursor Synthesis along the Sidewall of Pole-Growing Mycobacteria. Elife 2018, 7, e37243 10.7554/eLife.37243. 30198841 PMC6191288

[ref24] Yang M.; Jalloh A. S.; Wei W.; Zhao J.; Wu P.; Chen P. R. Biocompatible Click Chemistry Enabled Compartment-Specific PH Measurement inside *E. coli*. Nat. Commun. 2014, 5 (1), 4981 10.1038/ncomms5981. 25236616 PMC4174402

[ref25] Vitiello M.; Evangelista M.; Di Lascio N.; Kusmic C.; Massa A.; Orso F.; Sarti S.; Marranci A.; Rodzik K.; Germelli L.; Chandra D.; Salvetti A.; Pucci A.; Taverna D.; Faita F.; Gravekamp C.; Poliseno L. Antitumoral Effects of Attenuated Listeria Monocytogenes in a Genetically Engineered Mouse Model of Melanoma. Oncogene 2019, 38 (19), 3756–3762. 10.1038/s41388-019-0681-1. 30664692 PMC6756113

[ref26] Selvanesan B. C.; Chandra D.; Quispe-Tintaya W.; Jahangir A.; Patel A.; Meena K.; Alves Da Silva R. A.; Friedman M.; Gabor L.; Khouri O.; Libutti S. K.; Yuan Z.; Li J.; Siddiqui S.; Beck A.; Tesfa L.; Koba W.; Chuy J.; McAuliffe J. C.; Jafari R.; Entenberg D.; Wang Y.; Condeelis J.; DesMarais V.; Balachandran V.; Zhang X.; Lin K.; Gravekamp C. Listeria Delivers Tetanus Toxoid Protein to Pancreatic Tumors and Induces Cancer Cell Death in Mice. Sci. Transl. Med. 2022, 14 (637), eabc1600 10.1126/scitranslmed.abc1600. 35320003 PMC9031812

[ref27] Lepori I.; Oz Y.; Im J.; Ghosh N.; Paul M.; Schubert U. S.; Fedeli S. Bioorthogonal “Click” Cycloadditions: A Toolkit for Modulating Polymers and Nanostructures in Living Systems. Reactions 2024, 5 (1), 231–245. 10.3390/reactions5010010.

[ref28] Siegrist M. S.; Swarts B. M.; Fox D. M.; Lim S. A.; Bertozzi C. R. Illumination of Growth, Division and Secretion by Metabolic Labeling of the Bacterial Cell Surface. FEMS Microbiol. Rev. 2015, 39 (2), 184–202. 10.1093/femsre/fuu012. 25725012 PMC4462956

[ref29] Bird R. E.; Lemmel S. A.; Yu X.; Zhou Q. A. Bioorthogonal Chemistry and Its Applications. Bioconjug. Chem. 2021, 32 (12), 2457–2479. 10.1021/acs.bioconjchem.1c00461. 34846126

[ref30] Kuru E.; Hughes H. V.; Brown P. J.; Hall E.; Tekkam S.; Cava F.; de Pedro M. A.; Brun Y. V.; VanNieuwenhze M. S. In Situ Probing of Newly Synthesized Peptidoglycan in Live Bacteria with Fluorescent d-Amino Acids. Angew. Chem., Int. Ed. 2012, 51 (50), 12519–12523. 10.1002/anie.201206749. PMC3589519 23055266

[ref31] Liechti G. W.; Kuru E.; Hall E.; Kalinda A.; Brun Y. V.; VanNieuwenhze M.; Maurelli A. T. A New Metabolic Cell-Wall Labelling Method Reveals Peptidoglycan in Chlamydia Trachomatis. Nature 2014, 506 (7489), 507–510. 10.1038/nature12892. 24336210 PMC3997218

[ref32] Kirchenwitz M.; Stahnke S.; Prettin S.; Borowiak M.; Menke L.; Sieben C.; Birchmeier C.; Rottner K.; Stradal T. E. B.; Steffen A. SMER28 Attenuates PI3K/MTOR Signaling by Direct Inhibition of PI3K P110 Delta. Cells 2022, 11 (10), 1648 10.3390/cells11101648. 35626685 PMC9140127

[ref33] Rafelski S. M.; Theriot J. A. Mechanism of Polarization of Listeria Monocytogenes Surface Protein ActA. Mol. Microbiol. 2006, 59 (4), 1262–1279. 10.1111/j.1365-2958.2006.05025.x. 16430699 PMC1413586

[ref34] Ferraro N. J.; Kim S.; Im W.; Pires M. M. Systematic Assessment of Accessibility to the Surface of *Staphylococcus aureus*. ACS Chem. Biol. 2021, 16 (11), 2527–2536. 10.1021/acschembio.1c00604. 34609132 PMC9272369

[ref35] Kelly J. J.; Dalesandro B. E.; Liu Z.; Chordia M. D.; Ongwae G. M.; Pires M. M. Measurement of Accumulation of Antibiotics to *Staphylococcus aureus* in Phagosomes of Live Macrophages**. Angew. Chem., Int. Ed. 2024, 63 (3), e202313870 10.1002/anie.202313870. PMC10799677 38051128

[ref36] Kciuk M.; Gielecińska A.; Mujwar S.; Kołat D.; Kałuzińska-Kołat Ż.; Celik I.; Kontek R. Doxorubicin—An Agent with Multiple Mechanisms of Anticancer Activity. Cells 2023, 12 (4), 659 10.3390/cells12040659. 36831326 PMC9954613

[ref37] Nejadmoghaddam M.-R.; Minai-Tehrani A.; Ghahremanzadeh R.; Mahmoudi M.; Dinarvand R.; Zarnani A.-H. Antibody-Drug Conjugates: Possibilities and Challenges. Avicenna J. Med. Biotechnol. 2019, 11 (1), 3–23. 30800238 PMC6359697

[ref38] McCombs J. R.; Owen S. C. Antibody Drug Conjugates: Design and Selection of Linker, Payload and Conjugation Chemistry. AAPS J. 2015, 17 (2), 339–351. 10.1208/s12248-014-9710-8. 25604608 PMC4365093

[ref39] Caracciolo G.; Palchetti S.; Digiacomo L.; Chiozzi R. Z.; Capriotti A. L.; Amenitsch H.; Tentori P. M.; Palmieri V.; Papi M.; Cardarelli F.; Pozzi D.; Laganà A. Human Biomolecular Corona of Liposomal Doxorubicin: The Overlooked Factor in Anticancer Drug Delivery. ACS Appl. Mater. Interfaces 2018, 10 (27), 22951–22962. 10.1021/acsami.8b04962. 29905462

[ref40] Tentori P.; Signore G.; Camposeo A.; Carretta A.; Ferri G.; Pingue P.; Luin S.; Pozzi D.; Gratton E.; Beltram F.; Caracciolo G.; Cardarelli F. Fluorescence Lifetime Microscopy Unveils the Supramolecular Organization of Liposomal Doxorubicin. Nanoscale 2022, 14 (25), 8901–8905. 10.1039/D2NR00311B. 35719059

[ref41] Dimitrova D. S.; Berezney R. The Spatio-Temporal Organization of DNA Replication Sites Is Identical in Primary, Immortalized and Transformed Mammalian Cells. J. Cell Sci. 2002, 115 (21), 4037–4051. 10.1242/jcs.00087. 12356909

[ref42] Radoshevich L.; Cossart P. Listeria Monocytogenes: Towards a Complete Picture of Its Physiology and Pathogenesis. Nat. Rev. Microbiol. 2018, 16 (1), 32–46. 10.1038/nrmicro.2017.126. 29176582

[ref43] Salicrup L. A.; Ossandon M.; Prickril B.; Rasooly A. Bugs as Drugs, Potential Self-Regenerated Innovative Cancer Therapeutics Approach for Global Health. J. Glob. Health 2020, 10 (1), 010311 10.7189/jogh.10.010311. 32257138 PMC7100862

[ref44] Lou X.; Chen Z.; He Z.; Sun M.; Sun J. Bacteria-Mediated Synergistic Cancer Therapy: Small Microbiome Has a Big Hope. Nanomicro. Lett. 2021, 13 (1), 37 10.1007/s40820-020-00560-9. 34138211 PMC8187705

[ref45] Allemailem K. S. Innovative Approaches of Engineering Tumor-Targeting Bacteria with Different Therapeutic Payloads to Fight Cancer: A Smart Strategy of Disease Management. Int. J. Nanomed. 2021, 16, 8159–8184. 10.2147/IJN.S338272. PMC8687692 34938075

[ref46] Azzi S.; Hebda J. K.; Gavard J. Vascular Permeability and Drug Delivery in Cancers. Front. Oncol. 2013, 3, 211 10.3389/fonc.2013.00211. 23967403 PMC3744053

[ref47] MacDiarmid J. A.; Mugridge N. B.; Weiss J. C.; Phillips L.; Burn A. L.; Paulin R. P.; Haasdyk J. E.; Dickson K.-A.; Brahmbhatt V. N.; Pattison S. T.; James A. C.; al Bakri G.; Straw R. C.; Stillman B.; Graham R. M.; Brahmbhatt H. Bacterially Derived 400 Nm Particles for Encapsulation and Cancer Cell Targeting of Chemotherapeutics. Cancer Cell 2007, 11 (5), 431–445. 10.1016/j.ccr.2007.03.012. 17482133

[ref48] MacDiarmid J. A.; Amaro-Mugridge N. B.; Madrid-Weiss J.; Sedliarou I.; Wetzel S.; Kochar K.; Brahmbhatt V. N.; Phillips L.; Pattison S. T.; Petti C.; Stillman B.; Graham R. M.; Brahmbhatt H. Sequential Treatment of Drug-Resistant Tumors with Targeted Minicells Containing SiRNA or a Cytotoxic Drug. Nat. Biotechnol. 2009, 27 (7), 643–651. 10.1038/nbt.1547. 19561595

[ref49] Sagnella S. M.; Yang L.; Stubbs G. E.; Boslem E.; Martino-Echarri E.; Smolarczyk K.; Pattison S. L.; Vanegas N.; St. Clair E.; Clarke S.; Boockvar J.; MacDiarmid J. A.; Brahmbhatt H. Cyto-Immuno-Therapy for Cancer: A Pathway Elicited by Tumor-Targeted, Cytotoxic Drug-Packaged Bacterially Derived Nanocells. Cancer Cell 2020, 37 (3), 354–370.e7. 10.1016/j.ccell.2020.02.001. 32183951

[ref50] Kong M.; D’Atri D.; Bilotta M. T.; Johnson B.; Updegrove T. B.; Gallardo D. L.; Machinandiarena F.; Wu I.-L.; Constantino M. A.; Hewitt S. M.; Tanner K.; Fitzgerald D. J.; Ramamurthi K. S. Cell-Specific Cargo Delivery Using Synthetic Bacterial Spores. Cell Rep. 2023, 42 (1), 111955 10.1016/j.celrep.2022.111955. 36640333 PMC10009695

[ref51] Chandra D.; Jahangir A.; Quispe-Tintaya W.; Einstein M. H.; Gravekamp C. Myeloid-Derived Suppressor Cells Have a Central Role in Attenuated Listeria Monocytogenes-Based Immunotherapy against Metastatic Breast Cancer in Young and Old Mice. Br. J. Cancer 2013, 108 (11), 2281–2290. 10.1038/bjc.2013.206. 23640395 PMC3681012

[ref52] Mohamed M. M.; Sloane B. F. Multifunctional Enzymes in Cancer. Nat. Rev. Cancer 2006, 6 (10), 764–775. 10.1038/nrc1949. 16990854

[ref53] Leong M. L.; Hampl J.; Liu W.; Mathur S.; Bahjat K. S.; Luckett W.; Dubensky T. W.; Brockstedt D. G. Impact of Preexisting Vector-Specific Immunity on Vaccine Potency: Characterization of *Listeria monocytogenes* -Specific Humoral and Cellular Immunity in Humans and Modeling Studies Using Recombinant Vaccines in Mice. Infect. Immun. 2009, 77 (9), 3958–3968. 10.1128/IAI.01274-08. 19528221 PMC2737989

[ref54] Kim S. H.; Castro F.; Paterson Y.; Gravekamp C. High Efficacy of a Listeria-Based Vaccine against Metastatic Breast Cancer Reveals a Dual Mode of Action. Cancer Res. 2009, 69 (14), 5860–5866. 10.1158/0008-5472.CAN-08-4855. 19584282 PMC3127451

[ref55] de Boo S.; Kopecka J.; Brusa D.; Gazzano E.; Matera L.; Ghigo D.; Bosia A.; Riganti C. INOS Activity Is Necessary for the Cytotoxic and Immunogenic Effects of Doxorubicin in Human Colon Cancer Cells. Mol. Cancer 2009, 8 (1), 108 10.1186/1476-4598-8-108. 19925669 PMC2785770

