# Supplementary material for: Enhancing the Anticancer Activity of Attenuated Listeria monocytogenes by Cell Wall Functionalization with “Clickable” Doxorubicin
Source: ACS Chem Biol. 2024 Sep 24;19(10):2131–40. doi: 10.1021/acschembio.4c00250 (PMC11494506; doi:10.1021/acschembio.4c00250)
Supplement: Supplementary file 1 — cb4c00250_si_001.pdf [file cb4c00250_si_001.pdf]

SUPPORTING INFORMATION

Enhancing the anticancer activity of attenuated *Listeria monocytogenes* by cell wall functionalization with "clickable" doxorubicin

Irene Lepori[1,2,&,£], Marta Roncetti[1,2,3,&], Marianna Vitiello[1,2,#], Elisabetta Barresi[4,5], Raffaella De Paolo[1,2], Paolo Maria Tentori[6], Caterina Baldanzi[1,2], Melissa Santi[7], Monica Evangelista[1], Giovanni Signore[8,$], Lorena Tedeschi[1], Claudia Gravekamp[9], Francesco Cardarelli[7], Sabrina Taliani[4,5], Federico Da Settimo[4,5], M. Sloan Siegrist[10,11,*], Laura Poliseno[1,2,*]

[1]*Institute of Clinical Physiology, National Research Council (CNR-IFC), Pisa, 56124, Italy*

[2]*Oncogenomics Unit, Core Research Laboratory, ISPRO, Pisa, 56124, Italy*

[3]*University of Siena, Siena, 53100, Italy*

[4]*Department of Pharmacy, University of Pisa, Pisa, 56126, Italy*

[5]*CISUP-Center for Instrument Sharing, University of Pisa, Pisa, 56126, Italy*

[6]*Center for Nanotechnology Innovation @NEST, Istituto Italiano di Tecnologia, Pisa, 56126, Italy*

[7]*NEST-Scuola Normale Superiore, Istituto Nanoscienze, CNR (CNR-NANO), Pisa, 56126, Italy*

[8]*Fondazione Pisana per la Scienza ONLUS, San Giuliano Terme, 56017, Pisa, Italy*

[9]*Department of Microbiology and Immunology, Albert Einstein College of Medicine, New York, 10461, USA*

[10]*Department of Microbiology, University of Massachusetts, Amherst, 01003-9316, USA*

[11]*Molecular and Cellular Biology Graduate Program, University of Massachusetts, Amherst, 01003-9316, USA*

[&]equal contribution

*Corresponding authors:

Laura Poliseno, PhD
Oncogenomics Unit
Core Research Laboratory (CRL), ISPRO
Institute of Clinical Physiology (IFC), CNR
Via Moruzzi 1
56124 Pisa, Italy
Phone: +39 050 315 2780
Email: laura.poliseno@cnr.it, l.poliseno@ispro.toscana.it

M. Sloan Siegrist, PhD
Department of Microbiology
418 Morrill Science Center IVN
University of Massachusetts
639 North Pleasant Street
Amherst, MA 01003-9298
Email: siegrist@umass.edu

Fig 1

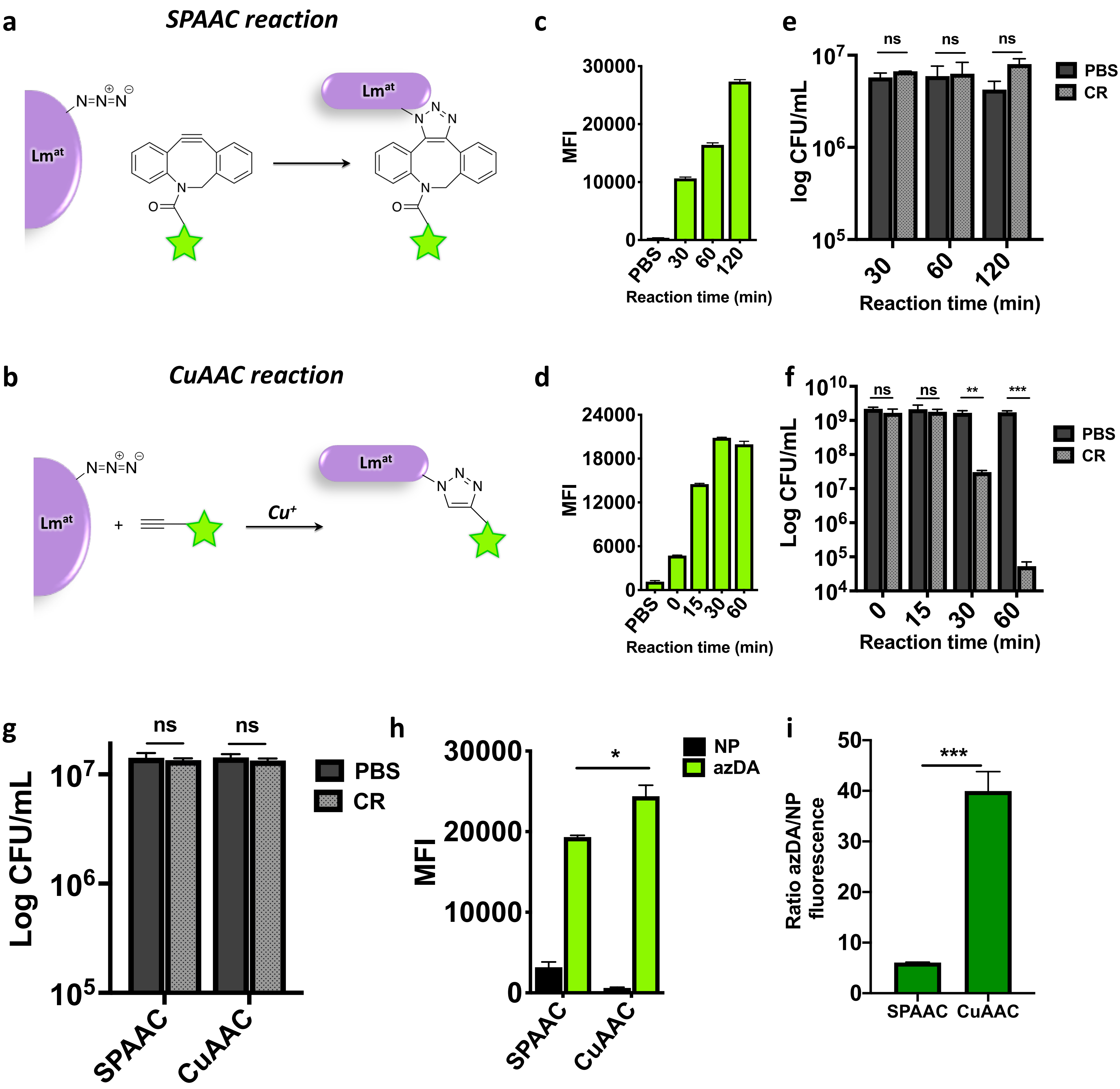

Supplementary Figure 1. Comparison of SPAAC and CuAAC reactions in terms of fluorescence intensity and viability of fluorescent Lm^{at}.

Loading efficiency was determined by flow cytometry analysis in terms of median fluorescence intensity (MFI), while toxicity was assessed by plating bacteria for colony forming units (CFU). Panels (**a**) and (**b**) show the chemical details of SPAAC and CuAAC reactions occurring on bacteria surface. In both cases, bacteria were metabolically labelled by overnight (ON) incubation with 2.5mM azDA probe. Then, click reaction was performed with alkyne-bearing AF488 green fluorophore as reaction partner.

(**c-f**) Establishment of the longest reaction time that is not toxic for bacteria, i.e. does not affect their viability. At the increase of incubation time, loading efficiency increases for both reactions (panel (**c**) and (**d**)). However, bacteria viability is preserved only in the case of SPAAC reaction (compare panel (**e**) and (**f**)).

(**g-i**) Comparison of loading efficiency at the longest reaction time that does not impair bacteria viability. At 60min reaction time for SPAAC and 15min reaction time for CuAAC, bacteria viability is totally preserved (**g**), while the loading efficiency is higher for CuAAC compared with SPAAC (**h**). This result becomes even more evident when the fluorescence acquired by bacteria upon click reaction is expressed as ratio over background (**i**).

NP: no probe; CR: click reaction (SPAAC or CuAAC depending on the graph).

Graphs show the mean±SEM of one experiment (three technical replicates), representative of three biological replicates. Unpaired t-test. *$p < 0.05$, **$p < 0.01$, ***$p < 0.001$. ns: not significant.

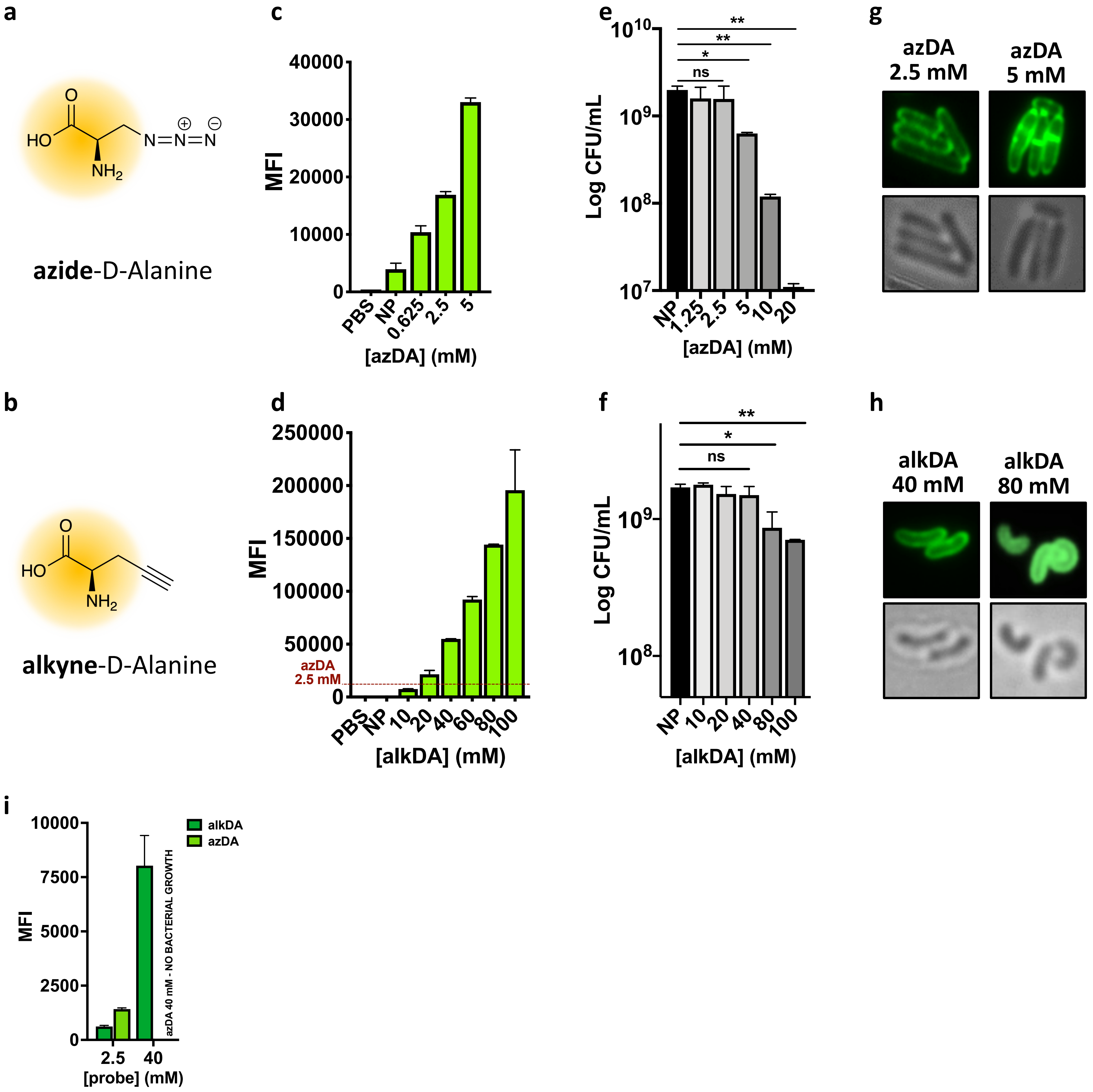

Supplementary Figure 2. Comparison of azido-D-Alanine and alkyne-D-Alanine probes in terms of fluorescence intensity and viability of fluorescent Lm^{at}.

Bacteria were metabolically labelled by ON incubation with azide-D-Alanine (azDA) (**a**) or alkyne-D-Alanine (alkDA) (**b**) probe. We opted for ON incubation to maximize the number of chemical handles available on Lm^{at} surface. Then, CuAAC reaction was performed with alk-AF488 or az-AF488 green fluorophore as reaction partner. Loading efficiency was determined by flow cytometry analysis in terms of median fluorescence intensity (MFI), while toxicity was assessed by plating bacteria for colony forming units (CFU).

For both probes, the increase in concentration results in increased loading efficiency (**c** and **d**), but it is also associated with decreased bacteria viability (**e** and **f**) and altered bacteria shape (**g** and **h**). azDA toxicity is stronger than that of alkDA, starting at 5mM (**e**), vs 80mM (**f**). The direct comparison of the highest non-toxic concentration for each probe (2.5mM for azDA and 40mM for alkDA, **i**) highlights that 2.5mM azDA probe leads to a higher fluorescence intensity compared to 2.5mM alkDA, but when the two probes are used at 40mM, the fluorescence intensity obtained with alkDA is higher than the one obtained with both probes at 2.5mM, while no bacterial growth is observed for azDA. NP: no probe.

Graphs show the mean±SEM of one experiment (three technical replicates), representative of three biological replicates. Unpaired t-test. *$p < 0.05$, **$p < 0.01$. ns: not significant.

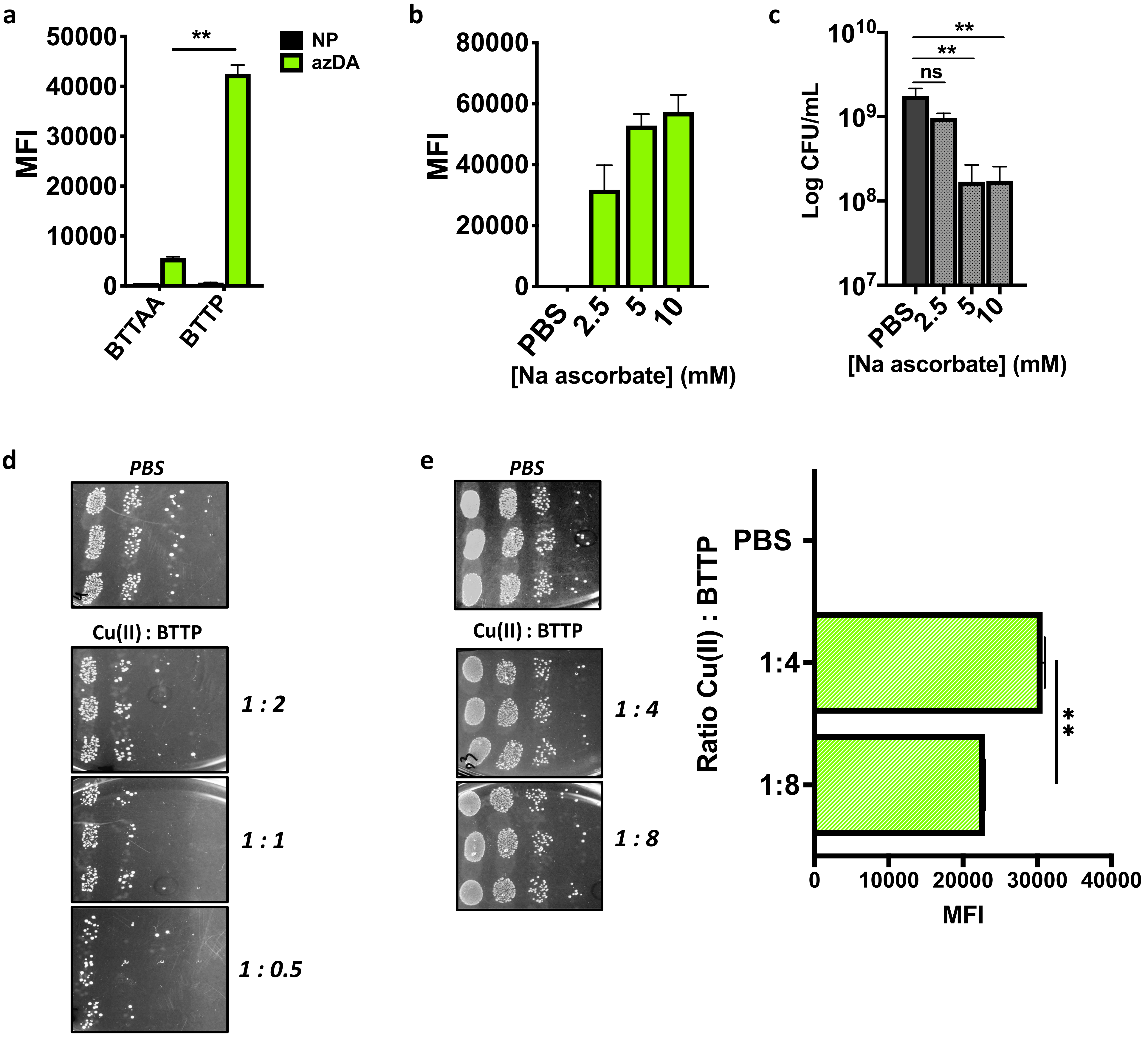

Supplementary Figure 3. Influence of CuAAC reaction components on fluorescence intensity and viability of fluorescent Lm^{at}.

Loading efficiency was determined by flow cytometry analysis in terms of median fluorescence intensity (MFI), while toxicity was assessed by plating bacteria for colony forming units (CFU).

(**a**) Comparison between BTTAA and BTTP as ligands. Bacteria were metabolically labelled with 2.5mM azDA probe, then alk-AF488 fluorophore was used as partner in the click reaction. The higher MFI indicates that BTTP ligand is superior at co-catalyzing CuAAC reaction, compared to BTTAA.

(**b-c**) Establishment of the optimal concentration of sodium ascorbate in the reaction mix. MFI (**b**) and viability (**c**) of Lm^{at} metabolically labelled with 40mM alkDA probe and then subjected to CuAAC reaction with az-AF488 fluorophore, using the indicated concentrations of sodium ascorbate in the reaction mix. Increasing the concentration of this salt, the loading efficiency increases, but viability decreases, probably due to the generation of increasingly high levels of Cu(I), the reduced toxic form of copper. The maximal concentration tolerated is 2.5mM.

(**d-e**) Establishment of the optimal $CuSO_4$:BTTP ratio in the reaction mix. Lm^{at} was metabolically labelled with 40mM alkDA probe and then subjected to CuAAC reaction with az-AF488 fluorophore. The concentration of $CuSO_4$ in the reaction mix was kept constant at 20µM (taken as 1), while that of BTTP was varied between 10µM (taken as 0.5) and 160µM (taken as 8). (**d**) Bacteria viability is strongly affected at low BTTP concentrations (1:0.5, 1:1 and 1:2 ratios), while it is unaffected at high BTTP concentrations, which seem to protect from copper toxicity (1:4 and 1:8 ratios, **e, left**). If fluorescence intensity is considered as well (**e, right**), 1:4 ratio results the optimal one.

NP: no probe.

Graphs show the mean±SEM of one experiment (three technical replicates), representative of three biological replicates. Unpaired t-test. **p < 0.01. ns: not significant.

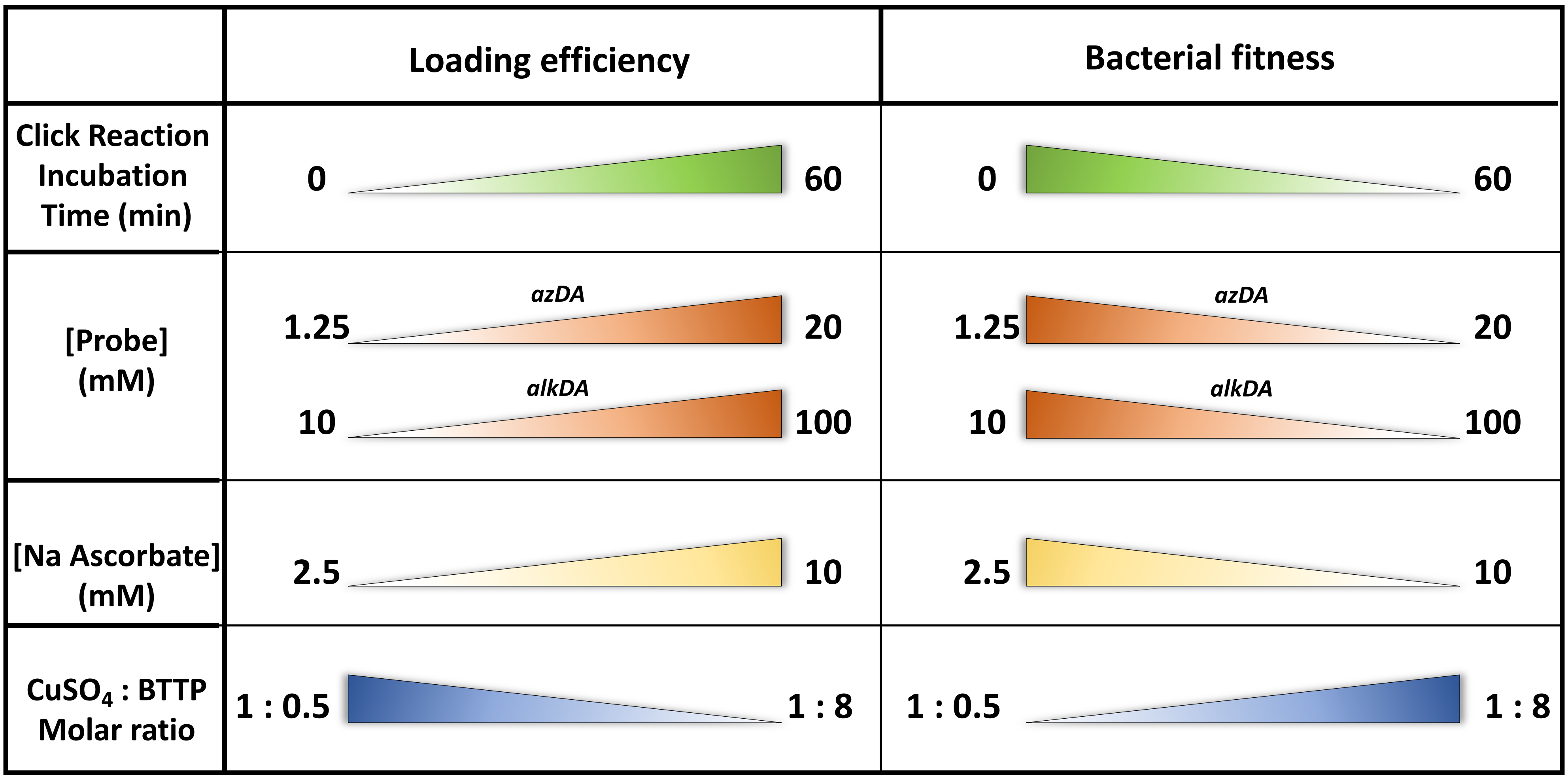

Supplementary Figure 4. Summary of the impact of click reaction conditions on loading efficiency and fitness of fluorescent Lm^{at}.

For all the investigated factors, the increase in loading efficiency is associated with a decrease in fitness (shape, viability, and proliferative activity) and *vice versa*. In the final protocol, we further optimized all the abovementioned factors, in order to achieve maximum loading efficiency without impairing bacteria fitness. Specifically, in the first step, Lm^{at} cell wall is metabolically labelled by ON incubation with 40mM alkDA. In the second step, the azide-bearing AF488 green fluorophore (25µM az-AF488) is attached to the cell wall *via* a CuAAC reaction composed of metabolically labelled bacteria at 7.5x10^8 CFU/mL, 2.5mM sodium ascorbate, 20µM $CuSO_4$ and 160µM BTTP, in PBS buffer, with incubation time set at 10min.

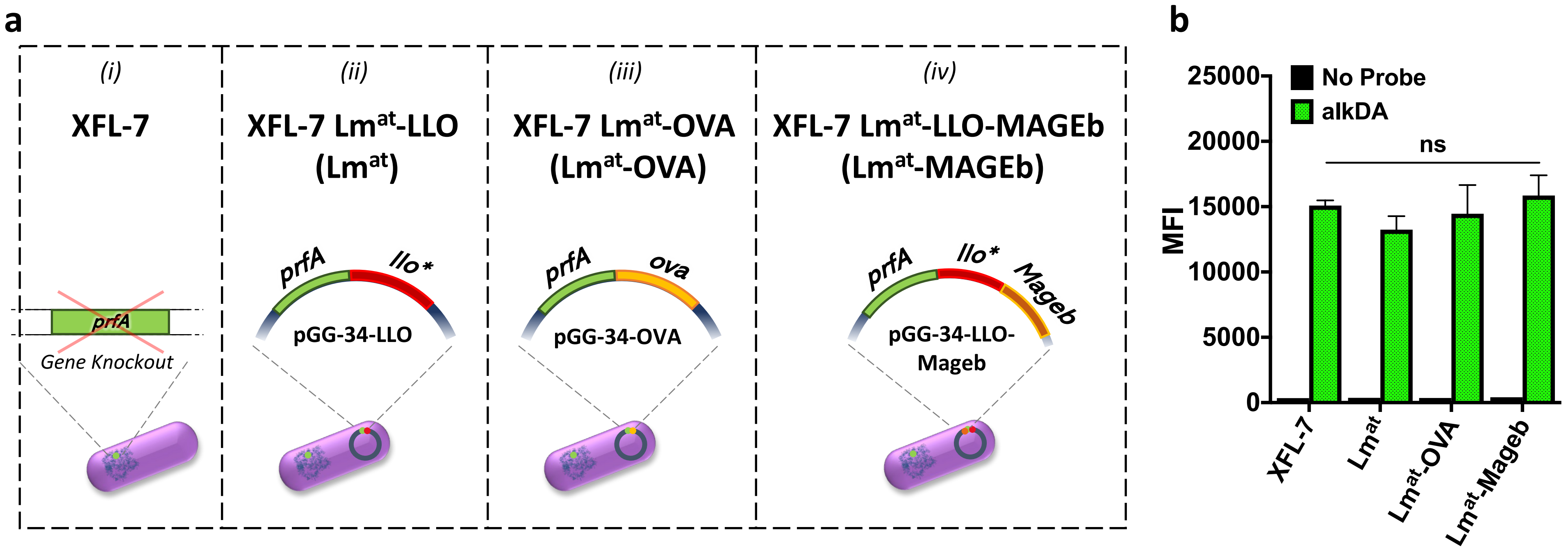

Supplementary Figure 5. The functionalization of Lm^{at} is not affected by genetic background.
(**a**) Strains of attenuated *Listeria monocytogenes* subjected to functionalization. The avirulent **XFL-7** strain (*i*) was obtained from 10403s strain of wt *Listeria monocytogenes* by knockout of the Positive Regulatory Factor A gene (*prfA*) (PMID: 11714814). PrfA is a transcription factor that regulates the expression of many virulent genes. Among them, Listeriolysin (LLO) is exploited by listeria to break free from cellular vacuoles and avoid digestion by lysosomes. The attenuated *Listeria monocytogenes* strain that we use, and that is widely employed as cancer vaccine, is in fact XFL-7 Lm^{at}-LLO strain (**Lm^{at}** for brevity, *ii*). Lm^{at} is obtained by transforming XFL-7 Lm^{at} strain with pGG-34-LLO plasmid. This plasmid drives the expression of PrfA, as well as that of a truncated version of Listeriolysin (LLO*), which can still mediate vacuole escape, but less efficiently compared to the wild type protein. The XFL-7 Lm^{at}-OVA strain (**Lm^{at}–OVA** for brevity, *iii*) is obtained by transforming XFL-7 strain with pGG-34-OVA plasmid, which expresses a fragment (aa 214-386) of chicken Ovalbumin instead of LLO*. This strain is avirulent, because unable to escape from vacuoles after host cell endocytosis (PMID: 30664692). Finally, **Lm^{at}-MAGEb** strain (*iv*), which is characterized by enhanced stimulation of the immune system against infected cancer cells, is obtained by transforming XFL-7 strain with pGG-34-LLO-MAGEb. This plasmid, together with LLO*, expresses a fragment (aa 311-660) of murine MageB Tumor Associated Antigen (PMID: 18728665).
(**b**) Cell wall functionalization of different Lm^{at} strains. The indicated strains of Lm^{at} were incubated ON with 40mM alkDA and then subjected to CuAAC reaction to obtain loading with AF488 green fluorophore. They all showed to be equally loaded, regardless of their genetic background. The graph represents the mean±SEM of at least three independent experiments. Unpaired t-test. ns: not significant.

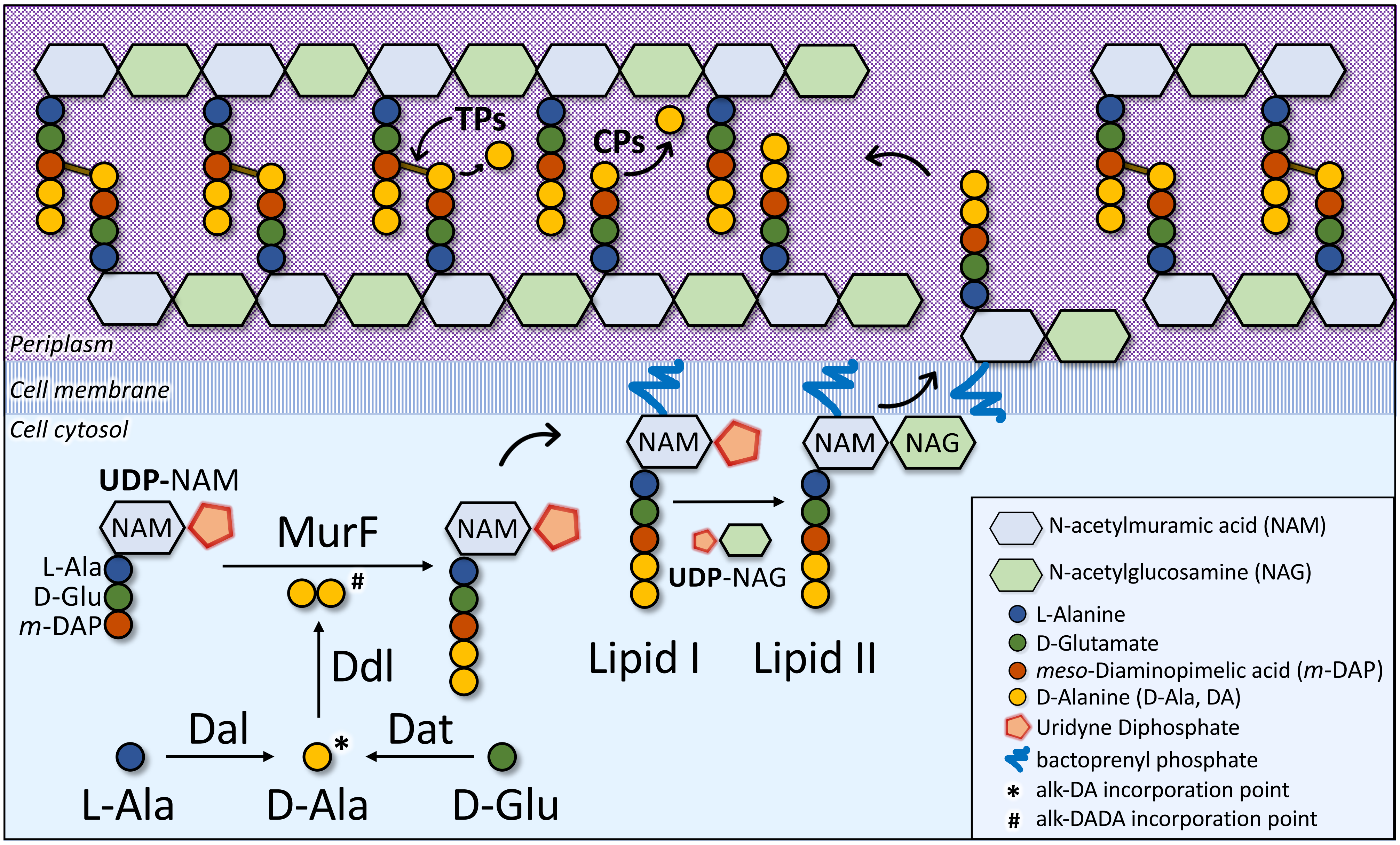

Supplementary Figure 6. Lm cell wall biosynthesis and points of alkDA and alkDADA probe incorporation.
The biosynthesis of Lm cell wall starts in the cytosol, where D-Alanine (D-Ala, DA) is generated from L-Alanine by D-Alanine racemase (Dal), or from D-Glutamate by D-amino acid aminotransferase (Dat). This is the main step where alkDA probe is incorporated. Then two D-Alanine are coupled by D-Alanyl-D-Alanine ligase (Ddl), to form D-Ala-D-Ala (DADA) dipeptide. This is the step where alkDADA probe is incorporated. Subsequently, UDP-N-acetylmuramoyl-tripeptide--D-alanyl-D-Alanine ligase (MurF) enzyme adds the DADA dipeptide to the pre-synthesized UDP-NAM-L-Ala-D-Glu-*m*-DAP unit, to generate the UDP-NAM-pentapeptide, which in turn is conjugated with bactoprenyl phosphate to generate Lipid I. At this point, UDP-NAG is added to Lipid I to generate Lipid II, which is flipped from the cytoplasmic to the periplasmic side of the cell membrane and employed to assemble the peptidoglycan (PG). Finally, remodelling enzymes cross-link the pentapeptide (a.k.a stem peptide) chains of one strand to the stem peptide chains of another strand, so that a 3D mesh-like layer is formed, which is strong and rigid. Specifically, D,D-carboxypeptidases (CPs, such as penicillin binding protein 5 (Pbp5)) remove the fifth D-Alanine of the stem peptide, while D,D-transpeptidases (TPs) remove the fifth D-Alanine of the stem peptide and cross-link the fourth D-Alanine to *meso*-Diaminopimelic acid.

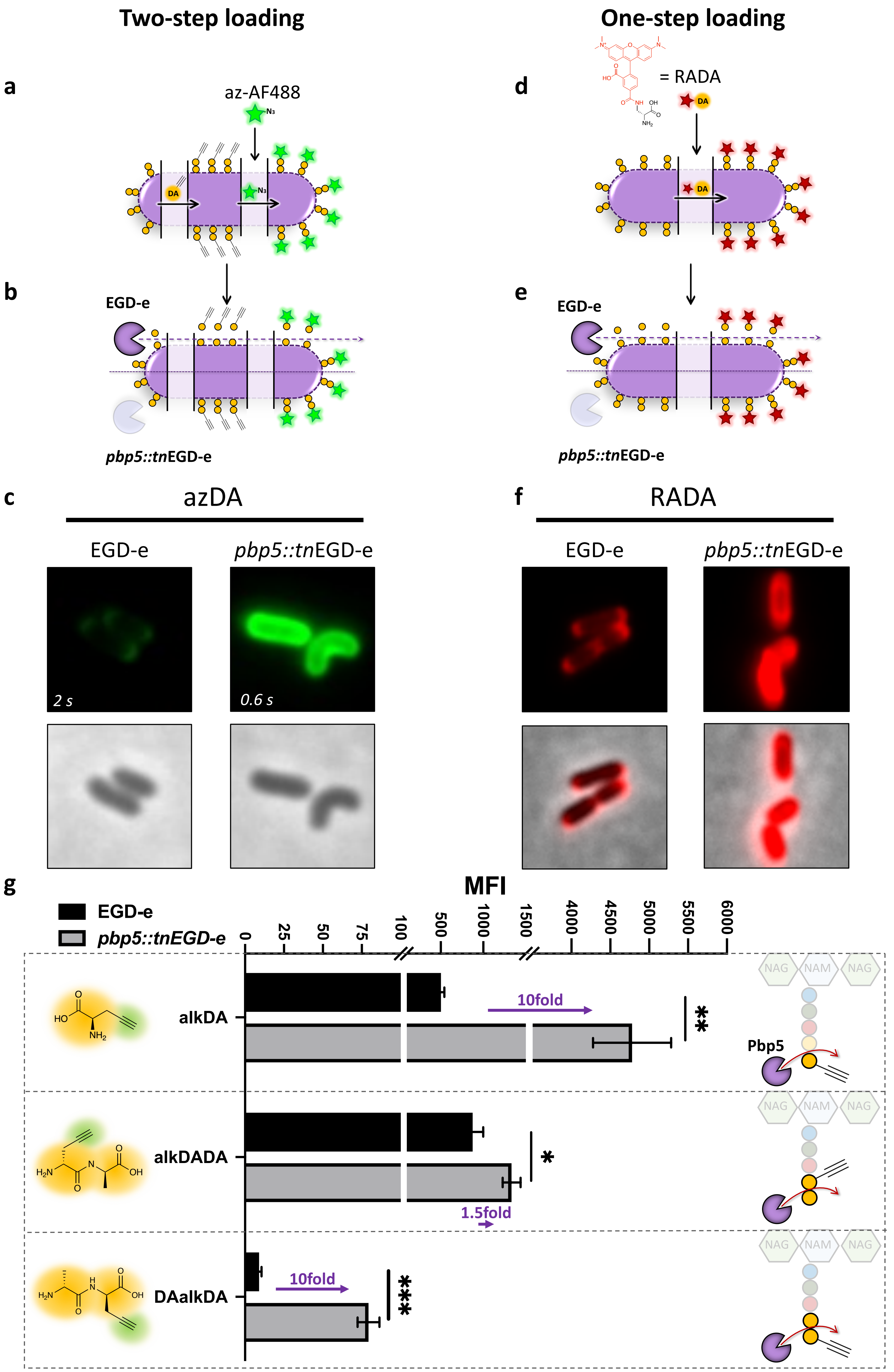

Supplementary Figure 7. Impact of the remodelling enzyme Pbp5 on *Listeria monocytogenes* cell wall functionalization.

(**a-c**) Impact of penicillin binding protein 5 (Pbp5) D,D-carboxypeptidase on the two-step loading approach using alkDA as probe. (**a**) Schematic representation of the two-step loading approach using alkDA as probe. (**b-c**) In the wt EGD-e Listeria strain (**b**, *upper*), Pbp5 removes the fifth D-Alanine of the stem peptide (yellow circle), resulting in decreased loading efficiency. Conversely, when the *pbp5::tn*EGD-e strain is used, which is deleted for Pbp5 (**b**, *lower*), the fifth D-Alanine is no longer removed and cell wall loading strongly increases. (**c**) Representative fluorescence microscope images of loaded EGD-e (*left*) and *pbp5::tn*EGD-e (*right*) strains. Images were taken at different exposure times (2sec for EGD-e and 0.6sec for *pbp5::tn*EGD-e) because of the strong difference in fluorescence intensity.

(**d-f**) Impact of Pbp5 enzyme on the one-step loading approach using RADA (rhodamine-D-Alanine) as probe. (**d**) Schematic representation of the one-step loading approach using RADA as probe. (**e-f**). The higher loading level of *pbp5::tn*EGD-e strain compared to wt EGD-e strain indicates that Pbp5 exerts a highly promiscuous activity and removes the fifth D-Alanine even when it carries bulky substituents on its side chain.

(**g**) Using alkDADA as probe, the impact of Pbp5 enzyme on the two-step loading approach is minimized. Metabolic labelling of wt EGD-e and *pbp5::tn*EGD-e Listeria strains was performed using alkDA (it incorporates in the <u>fifth</u> position of the stem peptide), alkDADA (it incorporates in the fourth and fifth position of the stem peptide and carries the alkyne group on the <u>fourth</u> D-Alanine), or DAalkDA (it incorporates in the fourth and fifth position of the stem peptide but carries the alkyne group on the <u>fifth</u> D-Alanine). Although basal incorporation varies from probe to probe and the cell wall of the mutant strain likely incorporates more probe because it is thicker (PMID: 16140473), the analysis of fluorescence intensity upon CuAAC reaction still indicates that Pbp5-mediated removal of the fifth D-Alanine is highly relevant only for alkDA and DAalkDA probes: the alk handle is expected to be incorporated on the fifth position of the stem peptide with both probes and, consistently, the MFI shown by the *pbp5::tn*EGD-e strain is much higher than that shown by the wt strain (~10fold). On the contrary, Pbp5-mediated removal of the fifth D-Alanine is much less impactful for alkDADA probe: the alk handle is expected to be incorporated on the fourth position of the stem peptide and, consistently, the MFI shown by the *pbp5::tn*EGD-e strain is just slightly higher than that shown by the wt strain (~1.5fold). The graph represents the mean±SEM of at least two independent experiments. Unpaired t-test. *$p < 0.05$, **$p < 0.01$, ***$p < 0.001$.

Fig 1

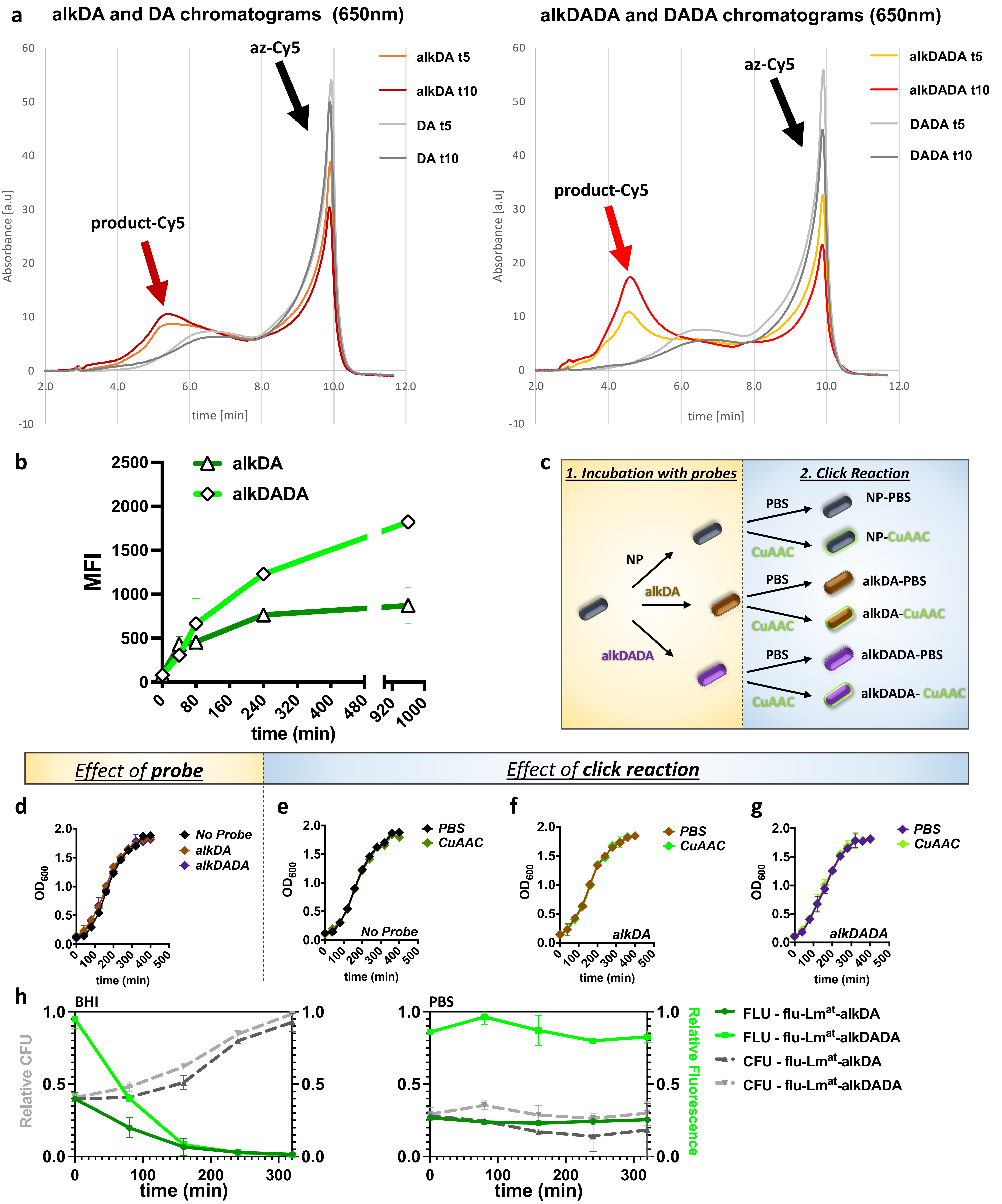

Supplementary Figure 8. Characterization of Lm^{at} functionalization with alkyne-D-Alanine (alkDA) and alkyne-D-Alanine-D-Alanine (alkDADA) probes.

(**a**) RP-HPLC analysis of the reactivity of alkDA probe (*left*) or alkDADA probe (*right*) with az-Cy5 fluorophore. A CuAAC reaction was set up in solution and the reaction mix was analyzed at different timepoints by reversed-phase (RP)-HPLC. Over time, a decrease in the size of az-Cy5 peak (black arrows) and a concomitant increase in the size of a peak with earlier elution time (red arrows) are observed, which is consistent with the reaction of the fluorophore with the probe. Probes with no alkyne group (DA and DADA, respectively) are used as negative controls (no peak with earlier elution time is observed).

(**b**) Fluorescence intensity of bacteria incubated with 40mM of alkDA or alkDADA probes for the indicated amount of time and then subjected to CuAAC reaction with az-AF488 fluorophore. Loading efficiency, determined by flow cytometry analysis in terms of median fluorescence intensity (MFI), is generally higher for alkDADA than alkDA. After ON incubation with the probes, a plateau is reached, which confirms that the maximal number of chemical handles have been incorporated on Lm^{at} surface.

(**c-g**) The optimized functionalization protocol does not affect Lm^{at} proliferation. (**c**) Schematic representation of the experimental set-up. In *Step 1*, Lm^{at} is incubated ON with 40nM alkDA or alkDADA. In *Step 2*, Lm^{at} from Step 1 is subjected to CuAAC reaction. (**d-g**) Proliferation of Lm^{at}, once subjected to the indicated experimental procedures. Refer to panel **c** for color coding.

(**h**) Loss of fluorescence by AF488-loaded Lm^{at}-alkDA and Lm^{at}-alkDADA over time. The loss of loading was investigated in two media: Brain Heart Infusion (BHI), which is the election growth medium for Lm^{at} (*left*), and the bacteriostatic PBS buffer (*right*). In BHI bacteria actively replicate (gray lines) and the loss of loading is fast (green lines). On the contrary, when bacteria are incubated in PBS, no proliferation is observed (gray lines), and, consequently, green fluorescence levels remain constant over time (green lines). These results suggest that replication is the main factor responsible for bacteria loss of loading over time. When alkDADA probe is used, fluorescence starts at higher level (see also **Figure 1b** and **panel b** above).

Graphs represent the mean±SEM of at least two independent experiments.

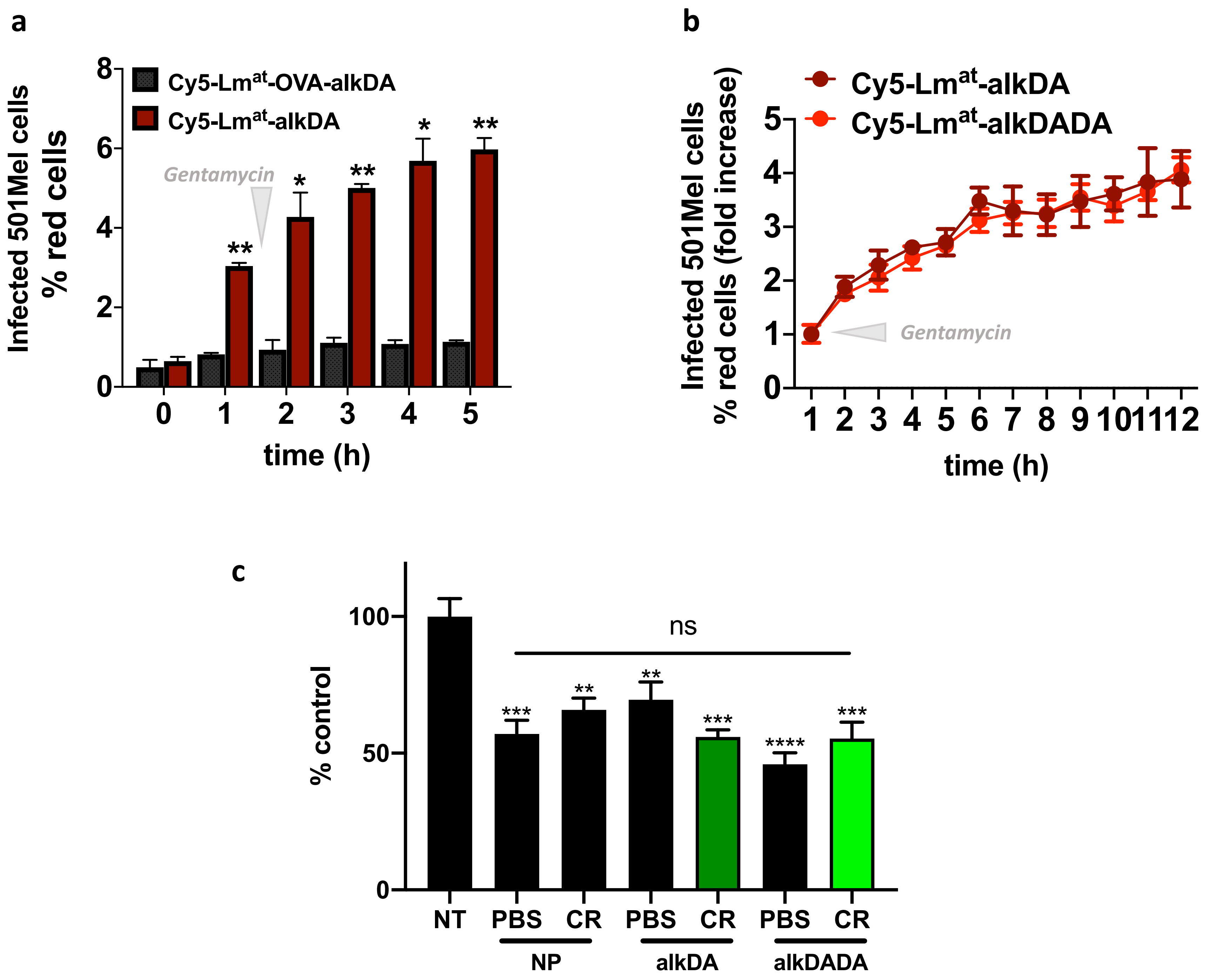

Supplementary Figure 9. Biological features of flu-Lm^{at}.

(**a-b**) Cell-to-cell spreading assay. As schematized in **Figure 1k**, 501Mel cells were infected at MOI 50. After 1h of infection, extracellular Lm^{at} was killed by medium replacement with fresh gentamycin-containing medium. Then, infected cells were collected at different time points post infection and directly analysed by flow cytometry to determine the percentage of cells that acquired red fluorescence due to listeria spreading. (**a**) Cell-to-cell spreading assay performed with Cy5-Lm^{at}-alkDA and Cy5-Lm^{at}-OVA-alkDA strains. Contrary to Cy5-Lm^{at}-OVA-alkDA strain, which is unable to spread because cannot escape from vacuoles after host cell endocytosis (grey bars), upon infection with Cy5-Lm^{at}-alkDA strain an increase in the percentage of red cells over time is detected (red bars). This result confirms that Lm^{at} spreading is the main reason why infected cells acquire red fluorescence, while the contribute of Lm^{at} turnover is minimal. (**b**) Cell-to-cell spreading assay performed with Cy5-Lm^{at}-alkDA and Cy5-Lm^{at}-alkDADA. Listeria shows similar spreading ability when metabolically labelled with both probes.

(**c**) Kill rate assay performed on A375 cells as reported in **Figure 1m** but using AF488-loaded Lm^{at} at MOI 200 instead of MOI 2000.

Graphs represent the mean±SEM of at least three independent experiments. Unpaired t-test. * $p < 0.05$, **$p < 0.01$, ***$p < 0.001$, ****$p < 0.0001$. ns: not significant.

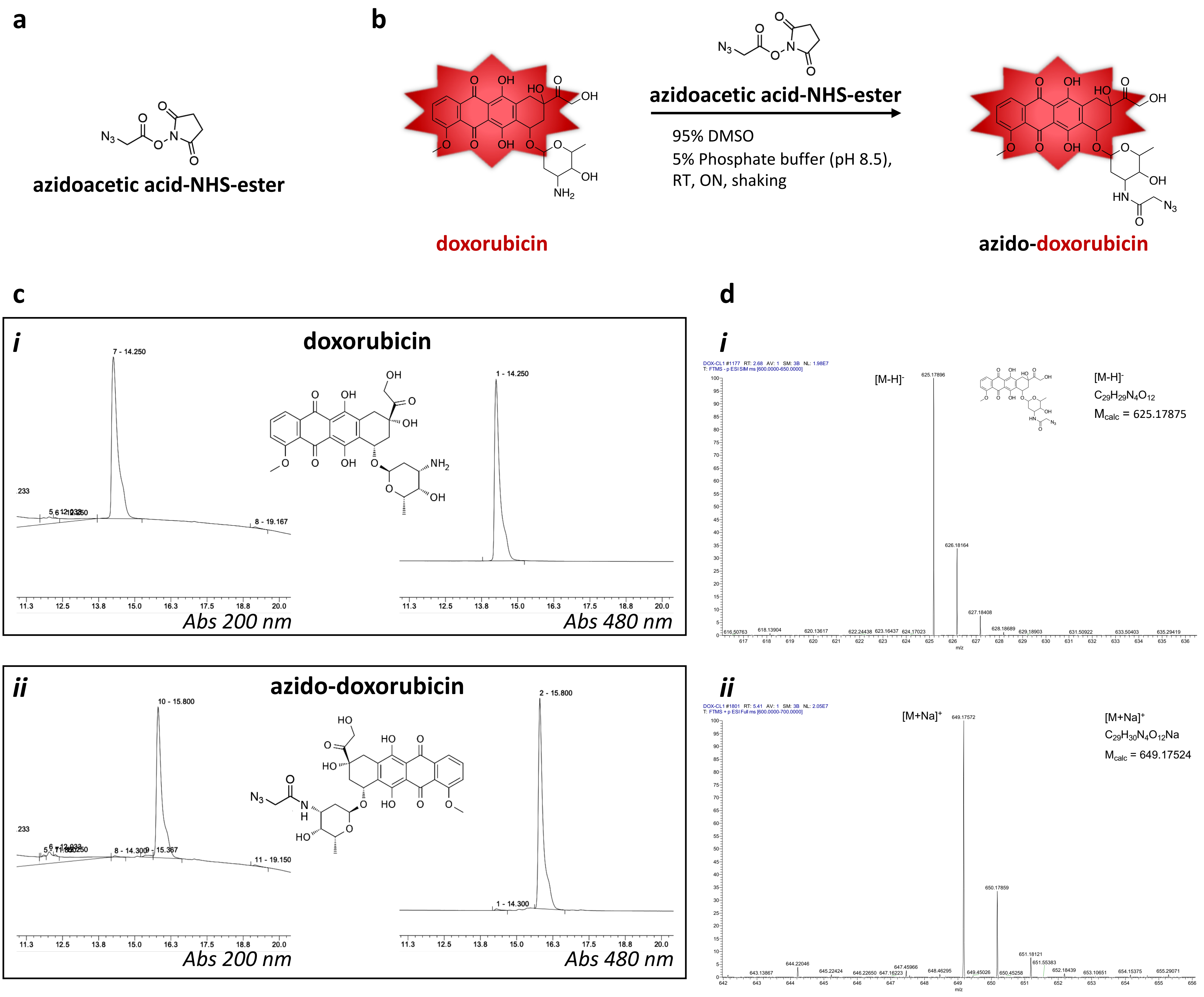

Supplementary Figure 10. Synthesis of azido-doxorubicin.

(**a**) Chemical structure of azidoacetic acid-N-hydroxysuccinimidyl (NHS)-ester.

(**b**) Schematic representation of the reaction conditions used to obtain azido-doxorubicin from doxorubicin and azidoacetic acid-N-hydroxysuccinimidyl (NHS)-ester.

(**c**) Chromatograms of doxorubicin (*i*) and azido-doxorubicin (*ii*), both at 200nm, to exclude the presence of non-fluorescent by-products (*left*), and at 480nm, the wavelength of maximum absorption of doxorubicin (*right*). Retention times: 14.250 minutes for doxorubicin and 15.800 minutes for azido-doxorubicin.

(**d**) High-Resolution Electron Spray Ionization mass spectrum of azido-doxorubicin in negative (*i*) and positive (*ii*) ion mode.

Fig 2

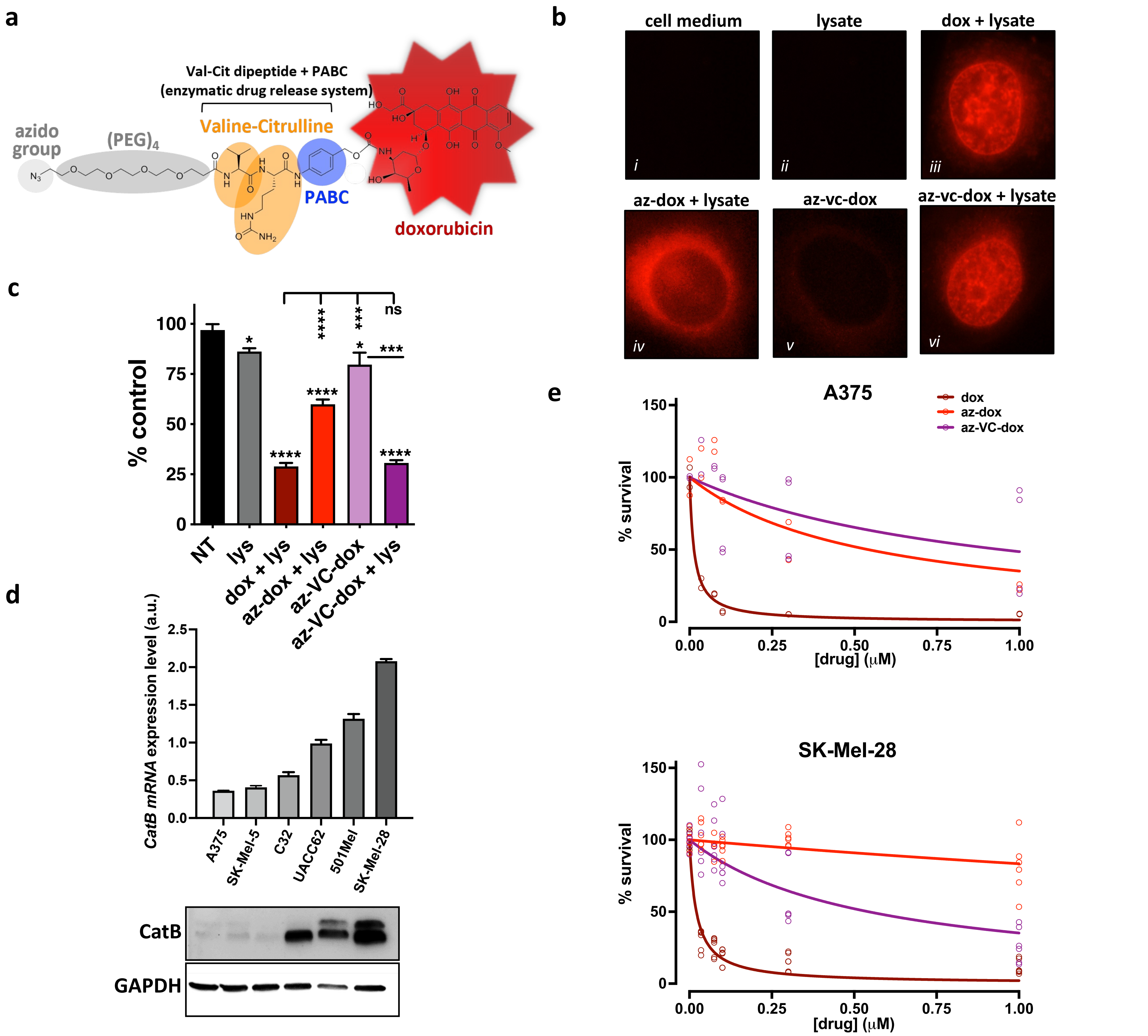

Supplementary Figure 11. Cathepsins-mediated cleavage of azido-Valine-Citrulline-doxorubicin and release of native drug in melanoma cell lines.

(**a**) Chemical structure of azide-PEG$_4$-Valine-Citrulline-PABC-doxorubicin (az-VC-dox). The compound contains: an azido group for click reaction with alkyne-labelled bacteria (light grey); a four units polyethylene glycol (PEG$_4$) spacer (grey) to increase molecule solubility; a Valine-Citrulline dipeptide (orange), which serves as drug release system in cancer tissues that overexpress Cathepsin enzymes; a *para*-aminobenzyl carbamate spacer (PABC, blue) directly attached to doxorubicin (red), *via* the amino group on the aminoglycoside portion of the drug. The PABC group acts as self-immolative spacer allowing the release of native doxorubicin after Cathepsin B cleavage of the Valine-Citrulline dipeptide.

(**b-c**) az-VC-dox cleavage by cellular Cathepsins restores fluorescence, nuclear localization, and cytotoxicity of doxorubicin. (**b**) Fluorescence microscope images of SK-Mel-28 melanoma cells incubated for 3h with: (*i*) cell medium; (*ii*) activated cell lysate from SK-Mel-28 cells; (*iii*) 3μM native dox in activated lysate; (*iv*) 3μM az-dox in activated lysate; (*v*) 3μM az-VC-dox in cell medium; (*vi*) 3μM az-VC-dox in activated lysate. Native doxorubicin can readily accumulate into cell nuclei. On the contrary, the drug loses this ability when functionalized on the primary amino group, both with small substituents like azidoacetic acid, and with the bulkier azido-Valine-Citrulline linker. The presence of the bulkier linker appears to quench red fluorescence as well. However, Cathepsins-mediated enzymatic cleavage of az-VC-dox fully restores doxorubicin fluorescence and nuclear localization, which is indicative of release in native form. Activation of Cathepsins present in cell lysate is induced by acidification up to pH4 (see Supplementary Methods for further details). If the lysate is kept at neutral pH, no cleavage of az-VC-dox is in fact observed (data not shown). The fluorescence and localization of native dox and az-dox in cell medium (data not shown) are indistinguishable from those displayed in activated lysate. (**c**) Kill rate assay performed on SK-Mel-28 cells treated for 48h with: cell medium (NT); activated cell lysate from SK-Mel-28 cells (lys); 0.3μM native dox in activated lysate (dox + lys); 0.3μM az-dox in activated lysate (az-dox + lys); 0.3μM az-VC-dox in cell medium (az-VC-dox); 0.3μM az-VC-dox in activated lysate (az-VC-dox + lys). Native doxorubicin is the most cytotoxic, while az-dox and especially az-VC-dox show reduced cytotoxicity. However, when az-VC-dox is incubated with the activated lysate, a full rescue in cytotoxicity is observed, which is consistent with the regain of nuclear localization (see panel **b**) and supports release in native form.

(**d-e**) Correlation between Cathepsin B levels and sensitivity to az-VC-dox. (**d**) Expression levels of Cathepsin B mRNA (qRT-PCR, *top*) and protein (western blot, *bottom*) in the indicated melanoma cell lines. (**e**) Survival curves of low Cathepsin B-expressing A375 melanoma cells (*top*) and high Cathepsin B-expressing SK-Mel-28 melanoma cells (*bottom*), treated with the indicated concentrations of doxorubicin (brown), az-dox (red) and az-VC-dox (purple) for 7 days. In the low Cathepsin B-expressing A375 melanoma cells, the cytotoxic effects of az-dox and az-VC-dox are very similar, while in the high Cathepsin B-expressing SK-Mel-28 melanoma cells az-VC-dox shows stronger cytotoxicity compared to az-dox, likely due to more efficient release of the native form of the drug through Cathepsin B-mediated cleavage.

Graphs represent the mean±SEM of at least three independent experiments. Unpaired t-test. *$p < 0.05$, ***$p < 0.001$, ****$p < 0.0001$. ns: not significant.

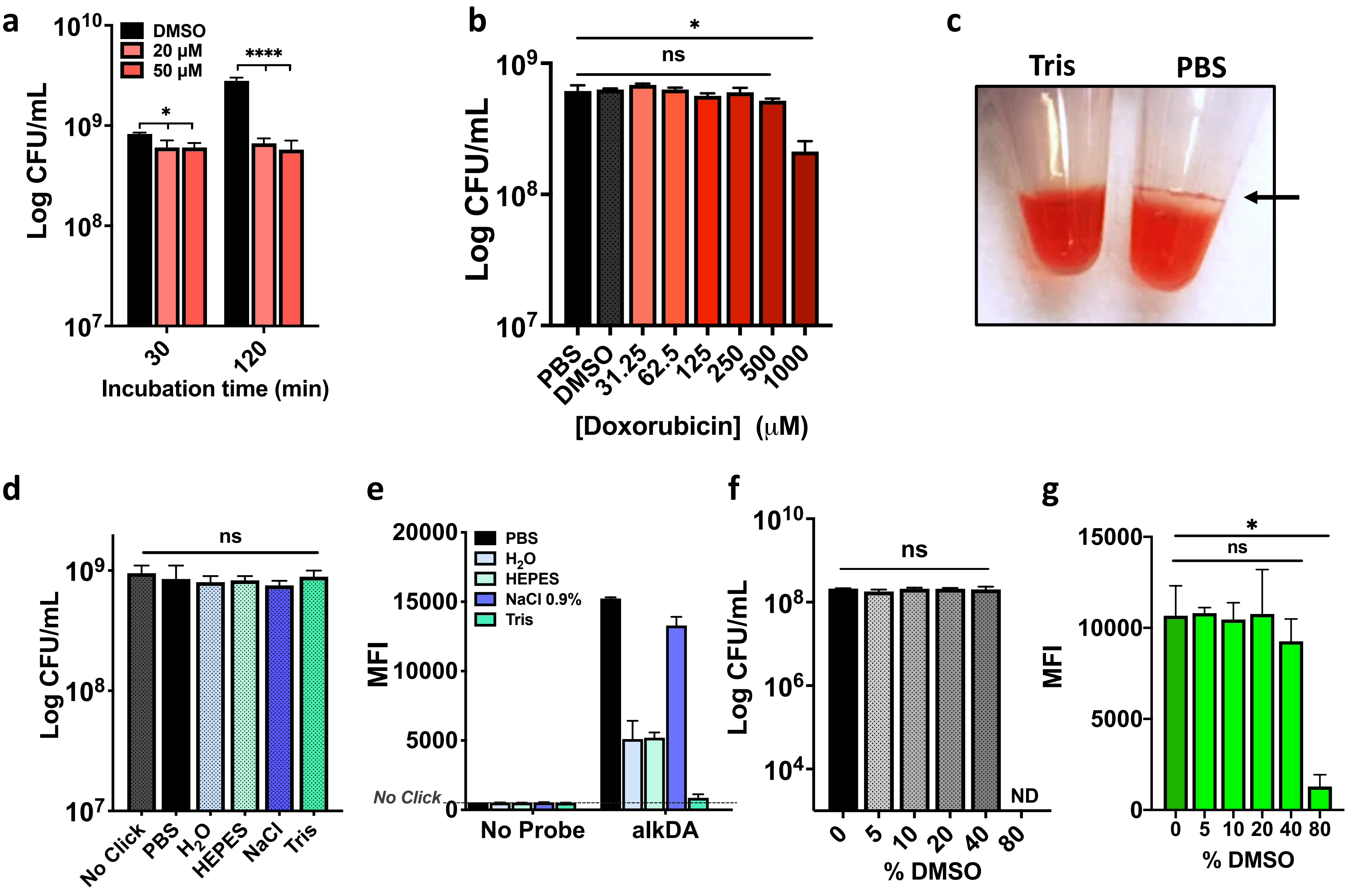

Supplementary Figure 12. Optimization of CuAAC reaction conditions for Lm[at] loading with az-dox and az-VC-dox.
(**a-b**) Evaluation of doxorubicin toxicity on Lm[at] in full medium (up to 120min, 37°C, BHI medium) (**a**), or in click reaction conditions (10min, room temperature, PBS buffer) (**b**). Doxorubicin is toxic for Lm[at] when incubated for a long time in BHI, which allows active replication. Conversely, when bacteria are kept for a short time in PBS, which is bacteriostatic, no toxic effect is observed even at very high dox concentrations (up to 500μM).
(**c**) Representative image of doxorubicin solubility/stability in Tris buffer (*left*) or PBS (*right*). The incubation of the drug in PBS (1mM, 2h, room temperature, shaking) leads to a cloudy suspension, with drug precipitates that are particularly evident on vial walls (arrow). Therefore, PBS cannot be used as buffer for CuAAC reaction.
(**d-g**) Viability and loading efficiency (measured as MFI) of Lm[at] incubated ON with 40mM alkDA probe and then subjected to CuAAC reaction, using az-AF488 as reaction partner and BTTP as ligand. Different solvents as alternatives to PBS and different concentrations of DMSO as co-solvent were tested. (**d-e**) When click reaction is performed in water, HEPES buffer, 0.9% w/v NaCl in water, or Tris buffer, Lm[at] viability is not affected (**d**). However, loading efficiency is comparable to that obtained with PBS only when 0.9% w/v NaCl in water is used (**e**). Tris buffer serves as negative control, as it is known to chelate copper ions and inhibit CuAAC (PMID: 22844652). (**f-g**) As co-solvent necessary for dox solubilization, DMSO does not affect Lm[at] viability (**f**) and loading efficiency (**g**), as long as its concentration is kept ≤40%.
MFI: median fluorescence intensity.
Graphs represent the mean±SEM of three independent experiments. Unpaired t-test. *$p < 0.05$, ****$p < 0.0001$. ns: not significant.

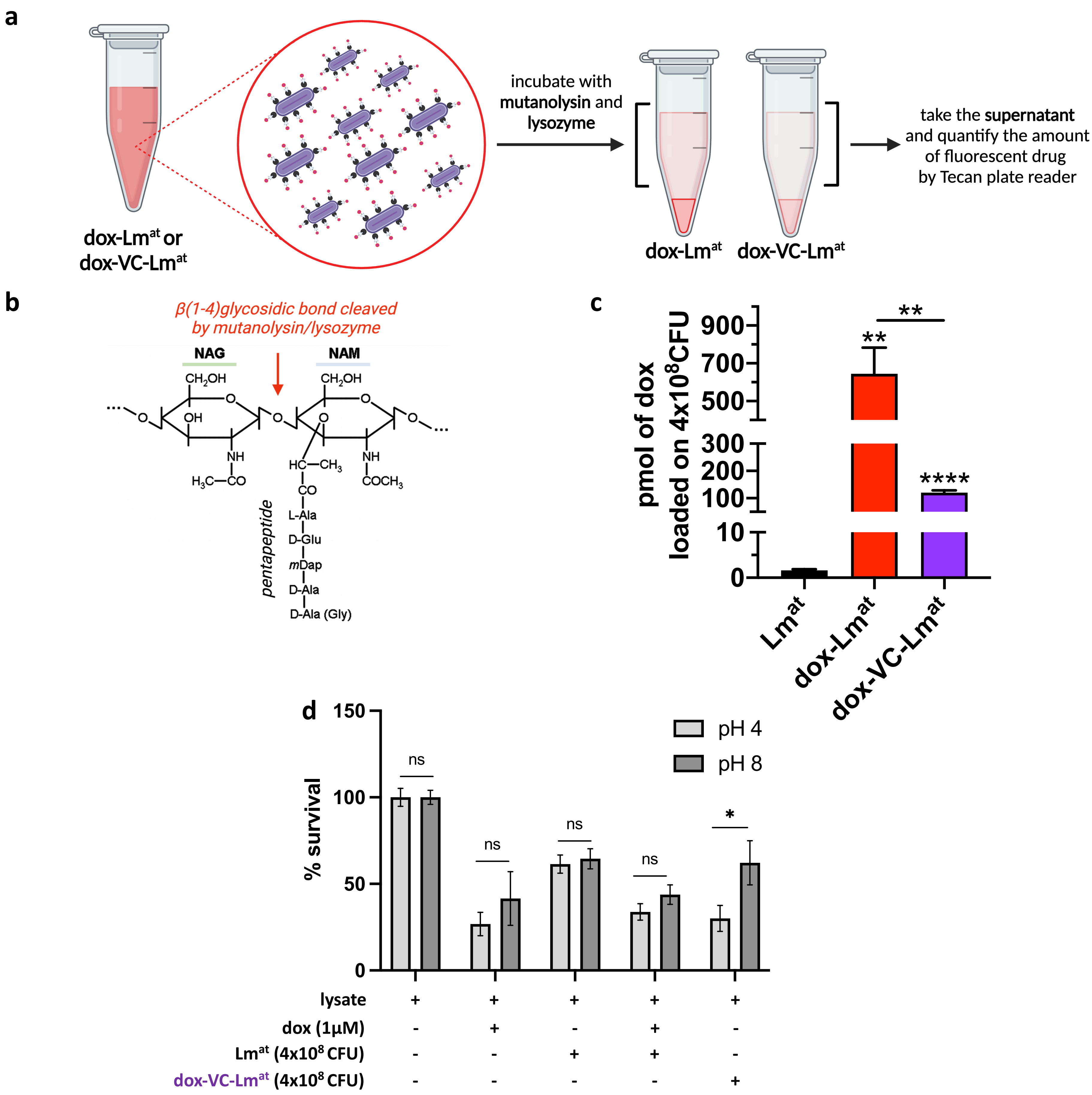

Supplementary Figure 13. Characterization of dox loaded on dox-Lm^{at} and dox-VC-Lm^{at}.

(**a-c**) Quantification of loaded drug by extraction from bacterial cell wall. (**a**) Representative cartoon of the experimental protocol used to extract dox from Lm^{at} cell wall and to quantify it. The incubation of dox-Lm^{at} and dox-VC-Lm^{at} with a mix of mutanolysin and lysozyme enzymes allows to digest bacterial cell wall, and to recover intact cargos in the supernatant. Then, the amount of dox present in the supernatant is quantified by fluorescence measurement. Cartoon created with Biorender.com. (**b**) Mechanism of action of mutanolysin and lysozyme. These enzymes selectively cleave the NAM-NAG bond within the bacterial peptidoglycan (see also **Suppl. Fig. 6**), causing the break down of cell wall. (**c**) Picomols of dox loaded on 4x10^{8}CFU of dox-Lm^{at} (red) dox-VC-Lm^{at} (purple).

(**d**) Cathepsin-mediated release of dox from dox-VC-Lm^{at} cell wall. The release is measured as a statistically significant decrease in SK-Mel-28 cell survival, upon 48h treatment with medium obtained from the incubation of 4x10^{8} CFU of dox-VC-Lm^{at} with the lysate of SK-Mel-28 cells, at pH8 (no activation of cellular Cathepsins, dark grey bar) vs pH4 (activation of cellular Cathepsins, light grey bar). Mock treatment with just the lysate is used as baseline. Treatment of SK-Mel-28 cells with lysate + 1μM dox is used as positive control of pH-insensitive decrease in cell survival. The decrease in cell survival observed by treatment of SK-Mel-28 cells with lysate + 4x10^{8} CFU of unloaded Lm^{at} is likely attributable to the fact that Listeria secretes virulence factors (PMID: 30504226) and they persist in the medium used to treat SK-Mel-28 cells. In any case, the decrease does not depend on pH.

Graphs represent the mean±SEM of at least two independent experiments. Unpaired t-test. * $p < 0.05$, ** $p < 0.01$, **** $p < 0.0001$. ns: not significant.

SUPPLEMENTARY METHODS

Reagents
Probes: alkyne-Dalanine (alkDA) (R-propargylglycine, Sigma-Aldrich or Acros Organics); 3-azido-D-alanine HCl ((R)-2-Amino-3-azidopropanoic acid hydrochloride, Jena Bioscience or Tocris Bioscience); EDA-DA and DA-EDA (custom made by WuXi AppTech); D-ala and D-ala-D-ala (Sigma-Aldrich). The pH of the aqueous probes solutions was checked before use and adjusted to 7 when necessary.
CuAAC reagents: $CuSO_4$ (Sigma-Aldrich); sodium ascorbate-L-(+) (Sigma-Aldrich); 3-(4-((bis((1-tert-butyl-1H-1,2,3-triazol-4-yl)methyl)amino)methyl)-1H-1,2,3-triazol-1-yl)propan-1-ol (BTTP) (Kumidas SA); BTTAA (Jena Bioscience).
Fluorophores: azide-AF488 (Jena Bioscience); alkyne-AF488 (Jena Bioscience); azide-Cy5 (Sigma-Aldrich); DBCO-AF488 (Jena Bioscience); azide-ATTO740 (ATTO-TEC GmbH).
Linkers: azidoacetic acid-N-hydroxysuccinimidyl (NHS) ester (Jena Bioscience); azide-PEG4-Valine-Citrulline-PABC-doxorubicin (Creative Biolabs).
Bacteria and cells growth media, supplements, and antibiotics: Brain Heart Infusion (BHI) (Sigma-Aldrich); Luria-Bertani (LB) (Sigma-Aldrich); Chloramphenicol (Sigma-Aldrich); DMEM High Glucose (HG), MEM and RPMI cell culture media (Euroclone); L-glutamine (Sigma-Aldrich); penicillin/streptomycin (Euroclone); gentamycin (Euroclone).
Drugs: doxorubicin hydrochloride (Sigma-Aldrich); SMER28 (Selleckchem).
Reagents for cell staining: paraformaldehyde (PFA) (Sigma-Aldrich); NOTOXhisto (Scientific Device Laboratory, Inc.); DAPI (4',6-Diamidino-2-Phenylindole, Dihydrochloride, Invitrogen); Fluoromount mounting glue (Bio-Optica).

alkyne-D-alanine-D-alanine, alkDADA
alkDADA (or EDA-DA) was synthesized as described in [1]. The recorded ^{1}H-NMR and ^{13}C-NMR spectra are in line with those reported in literature [1]. The purity of alkDADA was assessed by elementary analysis using Vario MICRO cube instrument (Elementar). Calculated for $C_8H_{12}N_2O_3 \cdot CF_3COOH$: C = 40.28; H = 4.39; N = 9.39. Experimental: C = 40.19; H = 4.68; N = 9.37.

Bacterial strains and culture conditions
Lm^{at}-LLO [2], Lm^{at}-OVA [2] and Lm^{at}-MAGEb [3] strains were grown at 37°C in BHI medium with 34µg/mL chloramphenicol. EGD-e and *pbp5::tn*EGD-e strains [4] were grown at 37°C in BHI medium without antibiotics.

Cell culture
Melanoma cell lines were grown at 37°C in a humidified atmosphere containing 5 % CO_2. A375, 501 Mel, SK-Mel-28 and SK-Mel-5 cells were cultured in DMEM HG; C32 cells were cultured in MEM; UACC62 cells were cultured in RPMI. All media were supplemented with 10 % foetal bovine serum (FBS), 2 mM L-glutamine, 100 U/mL penicillin, and 100 µg/mL streptomycin or without antibiotics when infected with Lm^{at}.

Statistical analyses
Data were analyzed with unpaired t-test (*in vitro* assays) or Kruskal-Wallis test (Dunn's multiple comparisons test, xenograft assay), using GraphPad Prism, GraphPad Software Inc. Statistical significance was set at $p<0.05$ (*p<0.05, **p<0.01, ***p<0.001, ****p<0.0001). The mean±SEM of at least three independent experiments is reported.

Methods used in Main Figures
Functionalization of Lm^{at} with a fluorophore through metabolic labelling and CuAAC click reaction
A single colony of Lm^{at} was inoculated in BHI +/- alkDA or alkDADA probes at 40 mM (or different concentrations when specified) and incubated overnight (ON, or shorter time points when specified) at 37°C in a shaking incubator. After OD_{600} normalization, metabolically labelled bacteria were washed three times in PBS to remove the excess of probe and

resuspended in PBS in half of the volume of CuAAC reaction mix: bacteria at 7.5x10^8 CFU/mL, 20 μM $CuSO_4$, 160 μM BTTP, 2.5 mM sodium ascorbate-L(+) (60 mM stock solution in water, freshly prepared), 25 μM azide-AF488 (or azide-Cy5 when specified). Chemical reagents were added one by one in this order and the solution was vortexed after each addition. After 10 min of incubation at room temperature in a shaking incubator, the reaction was stopped by adding an equal volume of 80 μM EDTA in PBS.
After click reaction, bacteria were washed three times in PBS, resuspended in PBS and analysed by flow cytometry (see below).
For stock preparation, bacteria where resuspended in DMEM HG, aliquoted, and stored at -80°C. The concentration of each stock was assessed by serial dilutions and plating for colony-forming units (CFU) on LB agar plates.

Functionalization of Lm^{at} with doxorubicin through metabolic labelling and CuAAC click reaction
A single colony of Lm^{at} was inoculated in BHI +/- 40 mM alkDADA and incubated ON at 37°C in a shaking incubator. After OD_{600} normalization, metabolically labelled bacteria were washed three times in PBS to remove the excess of probe and resuspended in physiological solution (0.9% w/v NaCl in water) plus 25% DMSO in one third of the volume of CuAAC reaction mix: bacteria at 7.5x10^8 CFU/mL, 60 μM $CuSO_4$, 480 μM BTTP, 7.5 mM sodium ascorbate-L(+) (60 mM stock solution in water, freshly prepared), 200 μM azide-doxorubicin or azide-valine-citrulline-doxorubicin (or the control azide-ATTO 740, 5 μM). Chemical reagents were added one by one in this order and the solution was vortexed after each addition. After 7 min of incubation at room temperature in a shaking incubator, the reaction was stopped by adding an equal volume of 160 μM EDTA in physiological solution.
After click reaction, bacteria were washed four times in physiological solution plus 10% DMSO, resuspended in DMEM HG, incubated for 10 min at 37°C in a shaking incubator (to better remove doxorubicin derivatives non-covalently attached to bacteria surface), then centrifuged, resuspended in PBS, and analyzed by visual inspection, flow cytometry and fluorescence microscopy (see below).
For stock preparation, bacteria where resuspended in DMEM HG, aliquoted, and stored at -80°C. The concentration of each stock was assessed by serial dilutions and plating on LB agar plates.

Flow cytometry analysis of loaded Lm^{at}
10000 events were acquired using BD Accuri™ C6 (BD) and Mean Fluorescence Intensity (MFI) was used as measure of labelling efficiency. Data were analysed using FlowJo software.

Fluorescence microscopy of loaded Lm^{at}
Bacteria prepared on agar pad were imaged with Nikon Eclipse E600 or Zeiss Axioscope A1 with 100x objectives.

Fluorescence Lifetime Imaging (FLIM) of dox-Lm^{at}
dox-Lm^{at} was mounted on a slide using antifade glue, dried ON at 4°C, and imaged. FLIM experiments were performed by using a Leica TCS SP5 confocal microscope (Leica Microsystems). Doxorubicin was excited at 470 nm by a pulsed diode laser operating at 40 MHz and using a 100X, 1,3NA objective. Doxorubicin signal was collected through the same objective and filtered in the 520-650 nm range by a photomultiplier tube linked with a time correlated single photon counting card and setup (PicoHarp 300, PicoQuant). Phasor analysis of lifetime data was performed using SimFCS software (Laboratory for Fluorescence Dynamics, University of California, Irvine).

Viability of Lm^{at} after incubation with probes and/or CuAAC click reaction
A single colony of Lm^{at} was inoculated in BHI +/- alkDA or alkDADA probes at 40 mM (or different concentrations when specified) and incubated ON at 37°C in a shaking incubator. Viability was assessed by serial dilutions and plating for CFU on LB agar plates. The same approach was used to determine Lm^{at} viability at the end of the CuAAC reaction.

Proliferative activity of loaded Lm^{at}
Frozen aliquots of differently labeled Lm^{at} stocks were diluted in BHI to a starting concentration of 5x10^{7} CFU/mL. Bacteria cultures were incubated in BHI at 37°C in a shaking incubator. Bacteria were collected at each time point and quantified by serial dilutions and plating for CFU on LB agar plates.

Infectivity and doubling time of loaded Lm^{at}
2.5x10^{4} A375 cells were seeded in 24-well plates and after 16 h listeria at MOI 100 was added to the cells. After 1h (for fluorescent Lm^{at}) or 2h (for doxorubicin-loaded Lm^{at}), the medium was replaced with fresh medium containing 50 μg/mL gentamycin. At 3h and 6h post-infection (for fluorescent Lm^{at}) or at 3h and 18h post-infection (for doxorubicin-loaded Lm^{at}), A375 cells were washed once with PBS and then hot (approx. 60°C) sterile water was added to lysate them. After 10 min incubation at 37°C, the concentration of intracellular bacteria was assessed by dilution and plating for CFU on LB agar plates.
In the case of SMER28 treatment, the infectivity protocol described above was modified as follows. 3.5x10^{4} A375 cells were seeded in 24-well plates and after 8h they were treated with or 50 or 200 μM SMER28 for 16h. The day after, listeria at MOI 100 was added to the cells. After 2h the medium was replaced with fresh medium containing 50 μg/mL gentamycin. At 3h post-infection, A375 cells were washed once with PBS and then processed as mentioned above.
Bacterial doubling time was calculated as the ratio of CFU at 6h vs 3h, using the Roth V. 2006 Doubling Time Computing Tool (http://www.doubling-time.com/compute.php).

Immunofluorescence of cells infected with loaded Lm^{at}
flu-Lm^{at}-alkDA and flu-Lm^{at}-alkDADA
10^{4} A375 cells were seeded in 24-well plates containing round coverslips and after 16h Lm^{at} at MOI 100 was added to the cells. After 1h, the medium was replaced with fresh medium containing 50 μg/mL gentamycin and cells were incubated for 2 additional hours. Then, cells were washed two times with PBS and fixed in PFA (4% in PBS) for 15 min at room temperature. After 3 washes in PBS, cells were incubated with blocking solution (1% BSA, 0.1% Triton X-100 in PBS) for 15 min at room temperature. Then, the solution was replaced with blocking solution containing a 1:5000 dilution of the primary anti-listeria antibody (Listeria O antiserum poly serotypes 1 and 4, #223021, BD) for 90 min at room temperature. After three 5-min washes with PBS, Alexa Fluor™ 647 Goat anti-Rabbit IgG (H+L) Highly Cross-Adsorbed Secondary Antibody secondary antibody (#A-21245, Thermo Fisher Scientific) was diluted 1:250 in blocking solution and was incubated for 1h at room temperature in the dark. The blocking solution of the secondary antibody also contained 300nM DAPI for nuclei staining in blue and 1:1000 Alexa Fluor™ 594 Phalloidin (#A12381, Thermo Fisher Scientific) for staining of actin filaments in red. After three 5-min washes with PBS, glasses were mounted on microscope slides using antifade glue (BioOptica) and let dry ON at 4°C.
Slides were visualized on a Leica SP2 confocal microscope equipped with an AOBS system using a 63Å~ oil immersion objective. Images were subsequently analyzed using ImageJ software. Regardless of staining with anti-Listeria antibody, the green channel was used for a direct visualization of Lm^{at} loaded with AF488 fluorophore.
dox-Lm^{at}
Compared to the protocol described above, actin filaments were stained in green using 1:1000 Alexa Fluor™ 488 Phalloidin (#A-12379, Thermo Fisher Scientific), while dox-Lm^{at} was visualized directly using the red channel.

Cell-to-cell spreading of fluorescent Lm^{at} (*protocol with anti-Listeria antibody*)
2,5x10^{5} 501 Mel cells were seeded in 6-well plates in complete DMEM HG w/o antibiotics. After 24h, cells were infected with MOI 50 of Lm^{at} that was previously labelled with alkDA or alkDADA probe and subjected to CuAAC click reaction with 25 μM azide-Cy5 fluorophore. After 2h of infection, the extracellular Lm^{at} was killed by replacing the medium with fresh DMEM HG medium containing 50 μg/mL gentamycin. At 3h and 18h post infection cells were washed with PBS, collected, and incubated with blocking solution (1% BSA, 0.001% Nonidet in PBS) containing 1:250 primary anti-Listeria antibody (Listeria O antiserum poly serotypes

1 and 4, #223021, BD) for 6h at 4°C. Cells were then washed twice in PBS and incubated in blocking solution with a 1:500 dilution of secondary antibody (Alexa Fluor™ 488 Goat anti-Rabbit IgG (H+L) Highly Cross-Adsorbed Secondary Antibody, #A-11034, Thermo Fisher Scientific) for 40 min at room temperature. Finally, cells were washed twice with PBS and the percentage of infected green cells was analysed by flow cytometry (CytoFLEX V2-B2-R0 Flow Cytometer (4 Detectors, 2 Lasers) B53017, Beckman Coulter). Data analysis was performed by FlowJo.

Kill rate of loaded Lm^{at}

10^3 A375 cells were seeded in 96-well plates in complete DMEM HG w/o antibiotics. After 16h cells were infected with MOI 2000 (or different MOI when specified) of Lm^{at} and after 3h the medium was replaced with fresh medium containing 50 μg/mL gentamycin. After 24h (for fluorescent Lm^{at}) or 48h (for doxorubicin-loaded Lm^{at}), cells were fixed with NOTOXhisto, according to the manufacturer's protocol, and stained with 300nM DAPI. Cells nuclei where then counted by fluorescent microscopy. 3 fields per well were visualized with Nikon EclipseTi2 fluorescent microscope equipped with a Nikon camera (Nikon NIS Elements software), 20x objective.

Immunofluorescence staining of MCM7 proliferation marker upon infection with doxorubicin-loaded Lm^{at}

$7x10^3$ A375 melanoma cells were seeded in 24-well plates containing round coverslips and 24 h later they were infected with dox-Lm^{at}, dox-VC-Lm^{at} or flu-Lm^{at} at MOI 1000. After 3h of infection, the medium was replaced with fresh medium containing 50 μg/mL gentamycin, and cells were incubated for additional 48 hours. Cells were then washed two times in PBS and fixed with 4% PFA in PBS for 10 min at room temperature. After 3 washes in PBS, cells were permeabilized by incubation with 0.5% Triton-X-100 in PBS for 10 min at room temperature. Cells were then washed three times in PBS and incubated in blocking solution (1% BSA in PBS) for 30 min at room temperature. Cells were then stained with a 1:50 dilution of anti-MCM7 mouse monoclonal primary antibody (clone 141.2, #sc-9966, Santa Cruz Biotechnology) in blocking solution ON at 4°C. After three washes with PBS, cells were incubated with a 1:500 dilution of the secondary antibody (Alexa Fluor™ 488 Goat anti-Mouse IgG (H+L) Highly Cross-Adsorbed Secondary Antibody, #A-11029, Thermo Fisher Scientific) in blocking solution in the dark for 30 min at room temperature. The blocking solution of the secondary antibody contained 300nM DAPI for nuclei staining in blue. Following three washes with PBS, the coverslips were mounted on a microscope slide by using Fluoromount mounting glue and let dry ON at 4°C.
Slides were visualized with Nikon EclipseTi2 fluorescent microscope equipped with a Nikon camera (Nikon NIS Elements software), 60x oil objective.

Zebrafish husbandry

The Zebrafish facility at CNR-IFC has been authorized by the Italian Ministry of Health (authorization #297/2012-A, issued on December 21, 2012) and by the Municipality of Pisa (authorization #DN-16/504, issued on June 7, 2013). Zebrafish (*Danio rerio*) experiments were carried out in accordance with the European Union guidelines for animal welfare [European Communities Council Directive of September 22, 2010 (2010/63/UE)]. All experimental protocols were approved by the Italian Ministry of Health (authorization #383/2020-PR).
Zebrafish are raised and maintained on a 14h/10h light/dark cycle at 28.5°C, in a zebrafish housing system (Tecniplast) under pH- and salinity-controlled conditions. Embryos are obtained by natural spawning, are maintained in E3 medium (5mM NaCl, 0.17mM KCl, 0.33mM CaCl2, 0.33mM MgSO4), and are staged according to hours post fertilization (hpf) and morphologic criteria [5]. Embryos euthanasia is performed by hypothermia shock for at least 20min, while adults are euthanized by exposure to excess of tricaine methanesulfonate (MS-222, #A5040, Merck).

Xenograft in zebrafish embryos

$1x10^6$ A375-PIG cells, which are stably infected with PIG-NotI (PIG) plasmid, hence stably express eGFP [6], were seeded in 100mm plates (1 plate per experimental condition) in complete

DMEM HG w/o antibiotics. After 24h, cells were infected with flu-Lm^{at} or dox-Lm^{at} at MOI 1000. After 2h, medium was replaced with fresh medium containing 50 μg/mL gentamicin. After an additional hour, infected cells were harvested using trypsin, counted, centrifuged and washed once with PBS. Then PBS was completely removed, and the tube was cooled down on ice. Cells were carefully resuspended in matrigel (Corning® Matrigel® Growth Factor Reduced (GFR) Basement Membrane Matrix, #354230) to reach the final concentration of 250 cells/nl (4ul of matrigel per 1x10^6 cells), by using cold pipette tips and avoiding bubble formation [7].

In the meantime, germ-free embryos of the *casper* line (ZDB-GENO-080326-11 line, kindly provided by Dr. Santoro, University of Padua, Italy) were generated as described in [8]. In brief, eggs were collected immediately after spawning and transferred to a sterile dish with sterilised E3 medium containing antibiotics (100 μg/mL ampicillin, 50 μg/mL gentamicin, 5 μg/mL kanamycin and 5 μg/mL amphotericin B). Embryos were monitored every 2h and unfertilized eggs were removed.

At 48hpf, embryos were manually dechorionated with forceps (Dumont No.5, #F6521-1EA, Sigma-Aldrich) and anaesthetised with 0.17 mg/mL tricaine (Sigma-Aldrich, #A5040). Cell suspension was loaded into a borosilicate glass capillary and 1nl (0,15mm, 250 cells) was injected into the yolk sac of the dechorionated embryos, using a microinjector (Tritech Research). After injection, embryos were allowed to grow in E3 medium for 48h at 34°C. In order to limit mortality caused by high bacterial load in the medium, embryos were washed twice a day with E3 medium supplemented with 50 μg/mL gentamicin. At least 100 embryos were injected per experimental condition.

Pictures of xenografts were acquired at 3 and 48h post-microinjection. Fluorescence imaging was carried out using Leica MZ10F stereomicroscope equipped with DFC3000 G camera and LAS X software version 3.7.0.20979. Area of cancer cell mass was measured using ImageJ software (http://rsb.info.nih.gov).

Xenograft experiments were performed in A375 cells for consistency with kill rate assays (**Fig. 1m and 2j**) and because we have extensively used them for this purpose [6 9 7]. In addition, xenograft experiments were performed only with dox-Lm^{at} for the following reasons. Kill rate assay indicates that dox-Lm^{at} and dox-VC-Lm^{at} have similar efficacy. Therefore, in compliance with the 3R principles, we opted for testing only one of the two types of dox-loaded *Listeria*. dox-Lm^{at} was chosen over dox-VC-Lm^{at} because it is less expensive (it does not contain the azide-PEG4-Valine-Citrulline-PABC-doxorubicin linker, which is a custom synthesis that we purchase from Creative Biolabs).

Methods used in Supplementary Figures

Comparison between SPAAC and CuAAC click reactions

A single colony of Lm^{at} was inoculated in BHI +/- azDA probe at 2.5 mM (or different concentrations when specified) and incubated ON at 37°C in a shaking incubator. After OD_{600} normalization, metabolically labelled bacteria were washed three times in PBS to remove the excess of probe and resuspended in PBS in half of the volume of SPAAC reaction mix or CuAAC reaction mix. SPAAC reaction mix: bacteria at 7.5x10^{8} CFU/mL, 25 μM DBCO-AF488 in PBS. CuAAC reaction mix: bacteria at 7.5x10^{8} CFU/mL, 200 μM $CuSO_4$, 800 μM BTTP (or BTTAA when specified), 2.5 mM (or other concentrations when specified) sodium ascorbate-L(+) (60 mM stock solution freshly prepared in water), 25 μM alkyne-AF488. Chemical reagents were added one by one in this order and the solution was vortexed after each addition. The reaction was incubated for 30 min (or different time when specified) at room temperature in a shaking incubator, then it was stopped by adding an equal volume of 80 µM EDTA in PBS.

After click reaction, bacteria were washed three times in PBS, resuspended in PBS and analysed by flow cytometry (10000 events, C6 Accuri^{TM}, BD).

Functionalization of wildtype EGD-e Lm and *pbp5::tn*EGD-e strains with a fluorophore through metabolic labelling and CuAAC click reaction

A single colony of wildtype EGD-e Lm or *pbp5::tn*EGD-e Lm was inoculated in BHI with alkDA, alkDADA or DAalkDA probes at 1 mM and incubated overnight (ON) at 37°C in a shaking incubator. After OD_{600} normalization, metabolically labelled bacteria were washed three times in PBS to remove the excess of probe and resuspended in half of the volume of CuAAC reaction mix: bacteria at 7.5x10^{8} CFU/mL, 20 µM $CuSO_4$, 160 µM BTTP, 2.5 mM sodium ascorbate-L(+) (60 mM stock solution in water, freshly prepared), 50 µM CalFluor 580 Azide (Vector Laboratories, #CCT-1371). Chemical reagents were added one by one in this order and the solution was vortexed after each addition. After 30-60 min at room temperature in a shaking incubator, the reaction was stopped by adding an equal volume of 80 µM EDTA in PBS.

After click reaction, bacteria were washed three times in PBS, resuspended in PBS and analysed by flow cytometry.

Functionalization of wildtype EGD-e Lm and *pbp5::tn*EGD-e strains with RADA probe

Wildtype EGD-e Lm and *pbp5::tn*EGD-e strains were incubated with 0.25 mM RADA at 37°C ON in BHI. The day after the culture was collected, washed three times with PBS and observed by fluorescence microscopy.

Reactivity of az-Cy5 with alkDA and alkDADA

To test the reactivity of az-Cy5 with alkDA and alkDADA, a click experiment was set up in solution and the reaction mix was analyzed at different timepoints by reversed-phase (RP)-HPLC. The CuAAC reaction mix was composed of: 100 µM alkDA/alkDADA, 25 µM azido-Cy5, 2,5 mM NaAsc, 20 µM CuSO4, 160 µM BTTP in PBS. In alternative, 100 µM DA/DADA probes (no alk handle) were used as negative controls. After 5-20 min at room temperature in a shaking incubator, the reaction was stopped by adding Sodium EDTA to 100µM. Reaction products were immediately subjected to a RP-HPLC run, using the following experimental conditions: Column LunaC8 Phenomenex; Mobil Phase Water/ACN 0,1% TFA; gradient from 0% to 100% ACN in 20′ starting from min 3; 20 µL injection.

Loss of fluorescence of Lm^{at} over time

Frozen aliquots of flu-Lm^{at}-alkDA and flu-Lm^{at}-alkDADA stocks were centrifuged and resuspended in BHI or PBS to obtain a concentration of 5x10^{6} CFU/mL. Bacteria suspensions were incubated for 320 min at 37°C in a shaking incubator. Every two doubling (80 min), aliquots of bacteria were collected, serially diluted, then plated for CFU on LB agar plates or analyzed by flow cytometry.

Cell-to-cell spreading assay of fluorescent Lm^{at} (*protocol without anti-Listeria antibody*)

3x10^{4} of 501 Mel cells were seeded in 96-well plates in DMEM HG + 10% FBS w/o antibiotics. After 16h, cells were infected with MOI 50 of Lm^{at} previously labelled with alkDA or alkDADA

probe and subjected to CuAAC click reaction with 25 µM azide-Cy5 fluorophore. After 1h of infection, the extracellular Lm^{at} was killed by replacing the medium with fresh DMEM medium containing 50 µg/mL gentamycin. At different time points cells were washed twice with PBS and collected. The percentage of infected red cells was analysed by flow cytometry (10000 events, BD Accuri™ C6, BD). Data analysis was performed by FlowJo.

Synthesis and analysis of azido-doxorubicin

Doxorubicin hydrochloride (Sigma-Aldrich) powder was resuspended in DMSO at a concentration of 1 mg/mL. Azidoacetic acid-N-hydroxysuccinimidyl (NHS) ester was resuspended with anhydrous DMSO in anhydrous atmosphere, aliquoted, and stored at -20°C. Reaction conditions: 2.6 mM doxorubicin, 52.5 mM azidoacetic acid-NHS ester in 95% DMSO and 5% phosphate buffer (50 mM, pH 8.5). The reaction was incubated for 16h at room temperature in a shaking incubator.

Purification and analysis were performed with reverse phase HPLC chromatography (Ultra-high-performance liquid chromatograph UltiMate® 3000, Dionex) on C18 column (Luna® 5 µm C8(2) 100 Å, LC Column 250 x 4.6 mm, Ea, part number 00G-4249-E0). Elution was performed at 1 mL/min. A 20 min linear gradient of acetonitrile, ranging from 25 to 75% in 0,1% trifluoroacetic acid (TFA) in water, was used. Wavelenghts: 200 nm, to exclude the presence of non-fluorescent by-products; 480 nm, which is the wavelength of maximum absorption of doxorubicin. Retention times: 14.250 min for doxorubicin; 15.800 min for azido-doxorubicin.

Azido-doxorubicin was also analyzed in negative and positive ion mode by High-Resolution Electron Spray Ionization mass spectrometry, using a Thermo LTQ Orbitrap XL mass spectrometer (Thermo Fisher Scientific).

Doxorubicin toxicity towards Lm^{at}

Doxorubicin toxicity on Lm^{at} was assessed both in proliferative and bacteriostatic conditions, the latter mimicking CuAAC reaction conditions.

To investigate doxorubicin toxicity in proliferative conditions, a single colony of Lm^{at} was inoculated in BHI and incubated ON at 37° C in a shaking incubator. The day after bacteria culture was diluted 1:3 in BHI containing 20 or 50 µM doxorubicin and incubated at 37°C in a shaking incubator. After 30 or 120 min Lm^{at} viability was assessed by serial dilutions and plating for CFU on LB plates.

To investigate doxorubicin toxicity in bacteriostatic conditions, after an overnight growth, bacteria culture was centrifuged and resuspended in PBS containing different concentrations of doxorubicin. After 10 min of incubation at room temperature in a shaking incubator, Lm^{at} viability was assessed by bacteria dilutions and plating for CFU on LB plates.

CuAAC click reaction in different solvents

A single colony of Lm^{at} was inoculated in BHI with 40 mM alkDA and incubated ON at 37°C in a shaking incubator. After OD_{600} normalization, metabolically labelled bacteria were washed three times in PBS to remove the excess of probe and resuspended in half of the volume of CuAAC reaction mix (10 µM $CuSO_4$, 80 µM BTTP, 1.25 mM freshly prepared sodium ascorbate-L(+), 25 µM azide-AF488) in different solvents: PBS, 20 mM Tris-HCl, 100 mM HEPES, physiological solution (0.9% w/v NaCl in water), water. Chemical reagents were added one by one in this order and the solution was vortexed after each addition. After 10 min of incubation at room temperature in a shaking incubator, the reaction was stopped by adding an equal volume of 160 µM EDTA. To determine loading efficiency, bacteria were washed three times in PBS, resuspended in PBS, and analysed by flow cytometry (10000 events, C6 Accuri™, BD). Bacteria viability was assessed by serial dilutions and plating for CFU on LB plates.

CuAAC click reaction at increasing concentrations of DMSO as co-solvent

A single colony of Lm^{at} was inoculated in BHI with 40 mM alkDA and incubated ON at 37°C in a shaking incubator. After OD_{600} normalization, metabolically labelled bacteria were washed three times in PBS to remove the excess of probe and resuspended in half of the volume of CuAAC reaction mix (10 µM $CuSO_4$, 80 µM BTTP, 1.25 mM fresh prepared sodium

ascorbate-L(+), 25 μM azide-AF488) containing increasing percentages of DMSO. Chemical reagents were added one by one in this order and the solution was vortexed after each addition. After 10 min of incubation at room temperature in a shaking incubator, , the reaction was stopped by adding an equal volume of 160 μM EDTA. To determine loading efficiency, bacteria were washed three times in PBS, resuspended in PBS, and analysed by flow cytometry (10000 events, C6 Accuri™, BD). Bacteria viability was assessed by serial dilutions and plating for CFU on LB plates.

Cleavage of azido-VC-dox using SK-Mel-28 cell lysate
Two 75 mL flasks were seeded with $2x10^6$ SK-Mel-28 cells each. After 48h, cells were collected by trypsinization and counted. $8x10^6$ cells were washed twice in 25 mL PBS and then centrifuged. The pellet was lysed in 3 mL of water, vortexed, and incubated for 15 min at 4°C in a shaking incubator. Then, to favor protein solubilization, 1.5 mL of acetone was added to the mixture and incubated for additional 15 min under the same conditions. To remove cell debris, the suspension was centrifuged at 7000 rcf for 10 min at 4°C. The supernatant was collected and concentrated in Speedvac to completely remove the acetone. Then, to activate cytoplasmic Cathepsins, the lysate was acidified by adding 0,2M HCl up to pH 4. The acidic lysate containing active Cathepsins was used to cleave 9μM az-VC-dox linker (3x concentration) by ON incubation at 37°C. The mixture was centrifuged to remove precipitated cell debris, then two volumes of complete DMEM medium were added to one volume of supernatant and the pH was adjusted to 8, adding 0,2 M NaOH if needed. Activated lysate was added to A375 cells seeded the day before on glass-bottom plates (CELLview™ Cell Culture Dish, four compartments, Thermo Fisher Scientific) at the density of 10^4 cells per compartment. Cells were imaged after 3h of incubation, using Nikon EclipseTi2 fluorescent microscope equipped with a Nikon camera (Nikon NIS Elements software), 60x oil objective.

mRNA expression levels of cathepsin B through qRT-PCR
Total RNA was extracted using QIAzol reagent (Qiagen), following the manufacturer's instructions. RNA was quantified using Nanodrop Lite (Thermo Fisher Scientific). 1 μg of RNA was retrotranscribed using QuantiTect Reverse Transcription Kit (Qiagen), following the manufacturer's instructions and using a S1000 Thermal Cycler (Bio-Rad). Genomic contamination of RNA was ruled out by PCR on the cDNA using PCR Master Mix (Thermo Fisher Scientific) and *ATPA1* primers [10].
Quantitative PCR was performed using SsoAdvanced Universal Supermix (Bio-Rad) on a CFX96 Real-Time System (Bio-Rad).
The reaction conditions were the following: 98 °C 30 s, (98 °C 3 s, 58 °C 20 s, 72 °C 10 s) ×40 cycles. In order to confirm the specificity of the reaction, a melting curve was performed after each PCR (from 65 °C to 95 °C with an increase of temperature of 0.5 °C/s). Reactions were performed in duplicate, and the average of the two Ct values was used to calculate the expression of the different transcripts by the $2^{-\Delta\Delta Ct}$ method, using the geometrical square mean of three housekeeping genes (*GAPDH*, *PBG*D, *SDHA*) as a reference [6].
PCR and qRT-PCR primers were purchased from Eurofins Genomics.
Cathepsin B primer sequences:
Forward primer (5'->3'): CTC TGA CCG GAT CTG CAT C
Reverse primer (5'->3'): TCA CAG GGA GGG ATG GAG TA

Protein extraction and western blot analysis
$4x10^5$ A375, $7.5x10^5$ C32 and UACC62, $5x10^5$ 501 Mel, $6.5x10^5$ SK-Mel-5 and SK-Mel-28 cells were seeded in p60 plates. As reported in[5], pellets were resuspended in 30 μl of lysis buffer (50 mM Tris-HCl, 1% TritonX100, 0.25% NaDeoxicholate, 1 mM PMSF, 2 mM Orthovanadate, proteinase inhibitor cocktail). The mixture was incubated for 30 min on ice, then sonicated for 30 min and finally centrifuged at 14000 rpm for 30 min at 4 °C. The supernatant was then quantified using Bradford reagent and the absorbance was read at 590 nm. The samples were heated for 5 min at 95 °C, separated on 10% SDS-polyacrylamide gels (Mini-PROTEAN Precast gel, Bio-Rad) and electrotransferred to polyvinylidene difluoride (PVDF) membranes using Trans-Blot Turbo system (Bio-Rad). Membranes were blocked at room temperature for 2 h using 3% BSA in TBST for the detection of Cathepsin B or 3% milk in TBST for the detection

of GAPDH. They were then incubated ON at 4 °C with the following primary antibodies: anti-Cathepsin B rabbit monoclonal antibody (#31718, Cell Signaling Technology, dilution 1:1000 in 3% milk in TBST); anti-GAPDH rabbit polyclonal antibody (#2118, Cell Signaling Technology, dilution 1:3000 in 3% milk in TBST). The detection of primary antibodies was performed using alkaline phosphatase-conjugated secondary antibodies and enhanced chemiluminescence reagents (Clarity Western ECL Substrate, Bio-Rad).

Growth curve assay with increasing concentrations of dox, az-dox, and az-VC-dox
Growth curve assays were performed as described in [11]. Briefly, $3x10^3$ A375 cells and $1.5x10^4$ SK-Mel-28 cells were seeded in 12-well plates (2 wells per drug concentration) and 24h later they were treated with different concentrations of the appropriate drug for a week. Cells were then fixed with 2% PFA and stained with crystal violet solution (0.1% crystal violet and 20% methanol in water). After the excess crystal violet solution was removed, the plates were washed with tap water and dried, then cells were de-stained using a 10% acetic acid solution. Absorbance of the resulting solution was read at 590 nm. Each sample was normalized on the vehicle-treated sample and the data were graphed as variation of cell percentage compared with samples treated with DMSO vehicle.

Extraction of doxorubicin from the cell wall of dox-Lm^{at}/dox-VC-Lm^{at} and quantification
$4x10^8$CFU bacteria were centrifuged (7650 *xg* for 20 min at 4°C) and resuspended in 500 μl of chilled TE buffer (50 mM Tris-HCl, 1 mM EDTA, pH 8) containing 1 mM PMSF. This step was repeated twice. The bacterial pellet was then resuspended in 170 μl of ice-cold mutanolysin/lysozyme mix and incubated for 2h at 37°C in a shaking incubator.
To obtain 1 mL of the mutanolysin/lysozyme mix in TES buffer (TES buffer is TE buffer with 20% (v/w) sucrose): 50 μl of 5.000 U/mL mutanolysin solution (Mutanolysin from Streptomyces globisporus, Sigma-Aldrich, #M9901), freshly prepared in chilled and filter-sterilized 0,1 M K_2HPO_4 solution at pH 6.2; 100 μL of 100 mg/ml lysozyme solution (Sigma-Aldrich), freshly prepared in TES buffer.
After incubation, the enzymatically digested bacteria were centrifuged at 14.000 *xg* for 5 min and the supernatant was collected. The amount of doxorubicin present in the supernatant was estimated by fluorescence measurement with Tecan Infinite M200 Pro plate reader (Ex480/Em590). To this end, a calibration curve composed of fourteen concentrations of doxorubicin, starting from 8 µM and performing a 1:2 serial dilution, were used.

Test of Cathepsins-mediated release of doxorubicin from dox-VC-Lm^{at}
Once SK-Mel-28 cell lysate was obtained as described above (see *Cleavage of azido-VC-dox using SK-Mel-28 cell lysate*), the pH was adjusted 8 by adding 0,2 M NaOH if needed, or it was acidified to 4 by adding 0,2M HCl. Acidic pH is necessary to activate cytoplasmic Cathepsins and promote the cleavage of the Valine-Citrulline dipeptide. Then, $4x10^8$ CFU of dox-VC-Lm^{at} were incubated in the lysate ON at 37°C. Further control samples included in the experiment were: lysate alone, lysate + 1 μM dox, lysate + $4x10^8$ CFU unloaded Lm^{at}, lysate + 1 μM dox + $4x10^8$ CFU Lm^{at}. Subsequently, the mixture was filtered with 3K and then 0,22 μM spin columns filters, to remove cellular debris as well as to make the solution sterile. Finally, two volumes of complete DMEM medium were added to one volume of supernatant and the pH was adjusted to 8, adding 0,2 M NaOH if needed. Activated lysate was added to SK-Mel-28 cells, seeded the day before at $3x10^3$ cells/96 well plate. After 48h of incubation, cells were fixed with NOTOXhisto, according to the manufacturer's protocol, and stained with 300nM DAPI. Cells nuclei where then counted by fluorescent microscopy. 3 fields per well were visualized with Nikon EclipseTi2 fluorescent microscope equipped with a Nikon camera (Nikon NIS Elements software), 20x objective.

REFERENCES

(1) Liechti, G. W.; Kuru, E.; Hall, E.; Kalinda, A.; Brun, Y. V.; VanNieuwenhze, M.; Maurelli, A. T. A New Metabolic Cell-Wall Labelling Method Reveals Peptidoglycan in Chlamydia Trachomatis. *Nature* **2014**, *506* (7489), 507–510. https://doi.org/10.1038/nature12892.

(2) Vitiello, M.; Evangelista, M.; Di Lascio, N.; Kusmic, C.; Massa, A.; Orso, F.; Sarti, S.; Marranci, A.; Rodzik, K.; Germelli, L.; Chandra, D.; Salvetti, A.; Pucci, A.; Taverna, D.; Faita, F.; Gravekamp, C.; Poliseno, L. Antitumoral Effects of Attenuated Listeria Monocytogenes in a Genetically Engineered Mouse Model of Melanoma. *Oncogene* **2019**, *38* (19), 3756–3762. https://doi.org/10.1038/s41388-019-0681-1.

(3) Kim, S. H.; Castro, F.; Gonzalez, D.; Maciag, P. C.; Paterson, Y.; Gravekamp, C. Mage-b Vaccine Delivered by Recombinant Listeria Monocytogenes Is Highly Effective against Breast Cancer Metastases. *Br J Cancer* **2008**, *99* (5), 741–749. https://doi.org/10.1038/sj.bjc.6604526.

(4) Siegrist, M. S.; Whiteside, S.; Jewett, J. C.; Aditham, A.; Cava, F.; Bertozzi, C. R. (D)-Amino Acid Chemical Reporters Reveal Peptidoglycan Dynamics of an Intracellular Pathogen. *ACS Chem Biol* **2013**, *8* (3), 500–505. https://doi.org/10.1021/cb3004995.

(5) Kimmel, C. B.; Ballard, W. W.; Kimmel, S. R.; Ullmann, B.; Schilling, T. F. Stages of Embryonic Development of the Zebrafish. *Developmental Dynamics* **1995**, *203* (3), 253–310. https://doi.org/10.1002/aja.1002030302.

(6) Marranci, A.; Jiang, Z.; Vitiello, M.; Guzzolino, E.; Comelli, L.; Sarti, S.; Lubrano, S.; Franchin, C.; Echevarría-Vargas, I.; Tuccoli, A.; Mercatanti, A.; Evangelista, M.; Sportoletti, P.; Cozza, G.; Luzi, E.; Capobianco, E.; Villanueva, J.; Arrigoni, G.; Signore, G.; Rocchiccioli, S.; Pitto, L.; Tsinoremas, N.; Poliseno, L. The Landscape of BRAF Transcript and Protein Variants in Human Cancer. *Mol Cancer* **2017**, *16* (1). https://doi.org/10.1186/s12943-017-0645-4.

(7) Marranci, A.; Prantera, A.; Masotti, S.; De Paolo, R.; Baldanzi, C.; Podda, M. S.; Mero, S.; Vitiello, M.; Franchin, C.; Laezza, M.; Comelli, L.; Arrigoni, G.; Cervelli, T.; Del Pozzo, G.; Poliseno, L. PARP1 Negatively Regulates MAPK Signaling by Impairing BRAF-X1 Translation. *J Hematol Oncol* **2023**, *16* (1), 33. https://doi.org/10.1186/s13045-023-01428-2.

(8) Pham, L. N.; Kanther, M.; Semova, I.; Rawls, J. F. Methods for Generating and Colonizing Gnotobiotic Zebrafish. *Nat Protoc* **2008**, *3* (12), 1862–1875. https://doi.org/10.1038/nprot.2008.186.

(9) Pietrobono, S.; De Paolo, R.; Mangiameli, D.; Marranci, A.; Battisti, I.; Franchin, C.; Arrigoni, G.; Melisi, D.; Poliseno, L.; Stecca, B. P38 MAPK-Dependent Phosphorylation of Transcription Factor SOX2 Promotes an Adaptive Response to BRAF Inhibitors in Melanoma Cells. *Journal of Biological Chemistry* **2022**, *298* (9), 102353. https://doi.org/10.1016/j.jbc.2022.102353.

(10) Vitiello, M.; Tuccoli, A.; D'Aurizio, R.; Sarti, S.; Giannecchini, L.; Lubrano, S.; Marranci, A.; Evangelista, M.; Peppicelli, S.; Ippolito, C.; Barravecchia, I.; Guzzolino, E.; Montagnani, V.; Gowen, M.; Mercoledi, E.; Mercatanti, A.; Comelli, L.; Gurrieri, S.; Wu, L. W.; Ope, O.; Flaherty, K.; Boland, G. M.; Hammond, M. R.; Kwong, L.; Chiariello, M.; Stecca, B.; Zhang, G.; Salvetti, A.; Angeloni, D.; Pitto, L.; Calorini, L.; Chiorino, G.; Pellegrini, M.; Herlyn, M.; Osman, I.; Poliseno, L. Context-Dependent MiR-204 and MiR-211 Affect the Biological Properties of Amelanotic and Melanotic Melanoma Cells. *Oncotarget* **2017**, *8* (15). https://doi.org/10.18632/oncotarget.15915.

(11) Vitiello, M.; Evangelista, M.; Zhang, Y.; Salmena, L.; Pandolfi, P. P.; Poliseno, L. PTENP1 Is a CeRNA for PTEN: It's CRISPR Clear. *J Hematol Oncol* **2020**, *13* (1). https://doi.org/10.1186/s13045-020-00894-2.
